# Ancestral synteny shared between distantly-related plant species from the asterid (*Coffea canephora *and *Solanum *Sp.) and rosid (*Vitis vinifera*) clades

**DOI:** 10.1186/1471-2164-13-103

**Published:** 2012-03-20

**Authors:** Romain Guyot, Florent Lefebvre-Pautigny, Christine Tranchant-Dubreuil, Michel Rigoreau, Perla Hamon, Thierry Leroy, Serge Hamon, Valérie Poncet, Dominique Crouzillat, Alexandre de Kochko

**Affiliations:** 1 UMR DIADE, Evolution et Dynamique des Génomes, Institut de Recherche pour le Développement (IRD), BP 64501, 34394 Montpellier Cedex 5, France; 2 Nestlé R&D Center, 101 Avenue Gustave Eiffel, Notre Dame d'Oé, BP 49716, 37097 Tours Cedex 2, France; 3 CIRAD, UMR AGAP, Montpellier F-34398, France

**Keywords:** Comparative genomics, Synteny, Genome evolution, *Coffea*, *Vitis*, *Solanum*

## Abstract

**Background:**

Coffee trees (Rubiaceae) and tomato (Solanaceae) belong to the Asterid clade, while grapevine (Vitaceae) belongs to the Rosid clade. Coffee and tomato separated from grapevine 125 million years ago, while coffee and tomato diverged 83-89 million years ago. These long periods of divergent evolution should have permitted the genomes to reorganize significantly. So far, very few comparative mappings have been performed between very distantly related species belonging to different clades. We report the first multiple comparison between species from Asterid and Rosid clades, to examine both macro-and microsynteny relationships.

**Results:**

Thanks to a set of 867 COSII markers, macrosynteny was detected between coffee, tomato and grapevine. While coffee and tomato genomes share 318 orthologous markers and 27 conserved syntenic segments (CSSs), coffee and grapevine also share a similar number of syntenic markers and CSSs: 299 and 29 respectively. Despite large genome macrostructure reorganization, several large chromosome segments showed outstanding macrosynteny shedding new insights into chromosome evolution between Asterids and Rosids. We also analyzed a sequence of 174 kb containing the ovate gene, conserved in a syntenic block between coffee, tomato and grapevine that showed a high-level of microstructure conservation. A higher level of conservation was observed between coffee and grapevine, both woody and long life-cycle plants, than between coffee and tomato. Out of 16 coffee genes of this syntenic segment, 7 and 14 showed complete synteny between coffee and tomato or grapevine, respectively.

**Conclusions:**

These results show that significant conservation is found between distantly related species from the Asterid (*Coffea canephora *and *Solanum *sp.) and Rosid (*Vitis vinifera*) clades, at the genome macrostructure and microstructure levels. At the ovate locus, conservation did not decline in relation to increasing phylogenetic distance, suggesting that the time factor alone does not explain divergences. Our results are considerably useful for syntenic studies between supposedly remote species for the isolation of important genes for agronomy.

## Background

**Figure 1:**
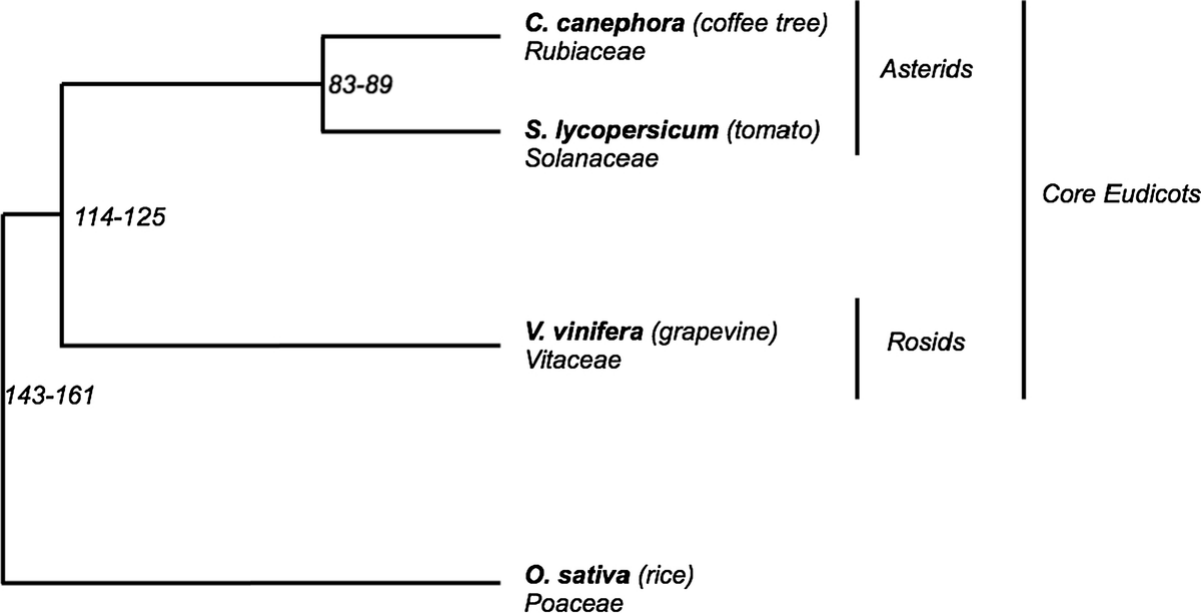
**Taxonomic relationships between *Solanum lycopersicum *(tomato, Solanaceae), *Coffea canephora *(coffee tree, Rubiaceae) and *Vitis vinifera *(grapevine, Vitaceae)**. The time scale of the divergence of the angiosperm families are indicated in millions of years, as published in Wikstrom et al. [2].

*Coffea *is a large genus that belongs to the Rubiaceae family, the fourth largest family of angiosperms, in term of species number. To date, this genus encompasses 103 perennial species, all native to Africa, Madagascar, the Mascarene Islands and the Comoros Islands [1]. It includes two economically important species: *C. arabica *L. and *C. canephora *Pierre, which represent a major agricultural commodity in world trade and one of the main sources of foreign exchange for Southern countries. The Rubiaceae is related to the Solanaceae family that contains numerous economically important crop species such as the tomato, the potato, the pepper, the eggplant, the tobacco and the petunia, all of these being annual. Both families belong to the Asterid I clade of dicotyledonous plants, and diverged from their common ancestor approximately 83-89 million years ago (MYA) (Figure 1) [2]. Besides phylogenetic considerations, Rubiaceae and Solanaceae are frequently considered as "sister" plant families based on genetic similarities observed between *C. canephora *(hereafter referred to as "coffee tree") and the tomato, *Solanum lycopersicum*, such as the genome size, 704 Mpb for the coffee tree [3] and 950 Mbp for the tomato [4], the basic chromosome number (11 and 12 for the coffee tree and the tomato, respectively), the cytogenetic chromosome architecture [5,6], the absence of polyploidization [7] and expressed gene repertoires in the seed and the cherry [8].

Based on available Expressed Sequences Tags (EST) databases [9], a large set of conserved single-copy genes, designated as putative orthologous genes or COSII, were selected *in silico *between Asterid plant species (eight species including *C. canephora *and *S. lycopersicum*) and the Rosid model plant *Arabidopsis thaliana *[7] . These COSII were used as valuable markers to construct genetic maps [10-12] to perform comparative mapping and to study chromosomal evolution in the Solanaceae [13]. Recently, using 257 COSII markers, a genetic map for the coffee tree was constructed and compared to that of the tomato [14]. Comparative mapping revealed that despite extensive rearrangements, a high level of conservation was detected between the coffee tree and the tomato, reinforcing the assessment of Rubiaceae and Solanaceae as "sister" plant families [14].

The Vitaceae family is another economically important family of angiosperms since it includes the *Vitis vinifera *species, known as the grapevine, a perennial plant, cultivated to be used as fruit or for beverage production. Recent phylogenetic analyses have placed the Vitaceae family as the earliest diverging lineage of the Rosid clade, which allows us to consider this family as the "sister" group of all other *Rosid *plant species [15]. Despite a fairly small genome size of about 475 Mbp [16] the presence of a high chromosome number (x = 19) suggested an ancestral polyploidy event of the grapevine genome [17]. Analysis of the draft sequence of the grapevine genome indicated both a complete absence of recent whole genome duplications and the contribution of ancient duplication events to the genome organization of the grapevine as well as to all of the Rosid species [18].

The grapevine and the coffee tree diverged from their last common ancestor approximately 114-125 MYA (Figure 1) [2], a long period of divergent evolution that should permit numerous chromosomal rearrangements to accumulate, allowing the genomes to reorganize significantly. So far, very few comparative mappings have been performed between very distantly related species belonging to two different clades, dicotyledonous or monocotyledonous [19-21]. Using genetic maps based on Expressed-Sequence Tags (EST) markers, four dicotyledonous crop species were compared to Arabidopsis, revealing common genome segments in a complex fragmented arrangement probably due to successive whole genome duplication in Arabidopsis [22]. In a pilot case study, Salse and coworkers [21] evaluated the synteny relationships between monocotyledonous and dicotyledonous species, which had diverged 146-161 MYA, using rice (*Oryza sativa*) and Arabidopsis genomes as models. In accordance with the relatively long period of evolution reinforced by successive whole genome duplications in both lineages, a very low level of synteny was observed between these two species.

Pairwise comparative mapping studies have been performed within the Solanaceae [12] and between the tomato and the coffee tree [8,14,23], all species belonging to the Asterid I clade. However, no multiple comparisons have been conducted using Solanaceae, the coffee tree and the distantly related grapevine species, from the basal Rosid clade, to detect synteny, or to study the pattern of chromosomal evolution between species that have not experienced recent polyploidization. Recently, extensive conservation of the microsynteny was described at the *EIN4 *locus between the coffee tree and the grapevine [23], suggesting that genome microstructure may be preserved over a long period of evolution. Such intriguing microsynteny raised the question of the extent of genome microstructure conservation and the possible presence of macrostructure conservation between the distantly related coffee tree and grapevine genomes.

Here, to better understand the structural relationships between coffee tree, tomato and grapevine genomes, and then to evaluate the genome conservation and evolution over the past 114-125 MY, we combined comparative mapping at the macro and micro-scale levels. Using a set of genetically mapped COSII sequences in the coffee tree and in the tomato, we identified numerous syntenic blocks in the grapevine genome sequence, providing evidence that segmental rearrangements have occurred since the divergence between the coffee tree and the tomato. Using a BAC clone sequence at the *ovate *locus, we investigated conservation at the macro- and micro-scale levels of the *ovate *region between Rubiaceae, Solanaceae and Vitaceae plant families.

## Methods

### Coffee mapping population

A COSII consensus reference genetic map of *C. canephora *was developed by Nestlé R&D (France) in collaboration with the Indonesian Coffee and Cocoa Research Institute (ICCRI) [14]. This map is derived from a segregating population of 93 F1 individuals issued from a cross between two highly heterozygous genotypes: a Congolese group genotype (BP409) and a Congolese-Guinean hybrid parent (Q121).

### Coffee genetic map

COSII markers were mapped into the coffee mapping population (BP409 × Q121) using three types of polymorphism: RFLP, SNP or SSR ([14] and MoccaDB, http://moccadb.mpl.ird.fr[24] for sequence, primer details, polymorphism and PCR conditions). The linkage analysis and map calculations were performed using JoinMap^® ^software version 4 [25,26] similarly as in Lefebvre-Pautigny et al. [14]. Genetic maps were drawn using MapChart software version 2.1 [27].

### Tomato mapping population

The tomato map used in this study (Tomato EXPAN-2000) is based on 80 F2 individuals from the cross *Solanum lycopersicum *LA925 × *Solanum pennellii *LA716 [12,13]. The tomato genetic map, used in this study and including all COSII markers is available at http://solgenomics.net/cview/map.pl?map_version_id=52.

### Grapevine (*Vitis vinifera*) data set

The grapevine reference genome sequences were downloaded from http://www.cns.fr/externe/GenomeBrowser/Vitis/ (Accessions: FN597015FN597047). Annotated coding sequences (CDS), representing 26,346 gene models were retrieved at the same location.

### Identification of orthologous sequences and syntenic blocks between coffee tree and grapevine and between tomato and grapevine

Coffee and tomato unigene sequences for each COSII loci were retrieved from the SOL Website http://solgenomics.net/ in April 2011. To identify orthologous COSII gene sequences based on sequence similarities between coffee (or tomato) COSII sequences and the grapevine genome, we performed BlastN and BlastX analyses. We used a BlastN with E-value significance thresholds of 10^-10 ^and 10^-6^. COSII sequences not identified in the grapevine genome were then used as a query for BLASTN searches against grapevine EST and BlastX searches using the successive E-value significance thresholds of 10^-30^, 10^-20 ^and 10^-5 ^against grapevine CDS. According to the results observed and based on a balance between sensibility and background, we selected a BlastN E-value of 10^-6 ^to identify orthologous COSII sequences. Sequences were considered as putative grapevine orthologs if only a single hit at a single position was found on the reference genome. A conserved syntenic segment (CSS) was defined by a minimum of three coffee or tomato COSII sequences that map to the same grapevine region at a maximal distance of 3,2 Mbp between pairs of markers. This genomic distance covers about 200 genes, when approximated by the *Vitis *genome size, 475 Mb [16] and the predicted number of genes, 30,434 http://www.cns.fr/externe/GenomeBrowser/Vitis/. For sequences anchored on the coffee genetic map, we selected a maximum genetic distance of 12 cM between pairs of syntenic markers, which correspond to about 1% of the coffee genetic map (1349 cM). The maximum distance for syntenic COSII sequences on the tomato genetic map was set to 14 cM, representing 1% of the tomato genetic map (1463 cM). This maximal distances were selected according to the methodology developed by Jung et al. [19], taking into account the phylogenic distances between our study species and genome coverage data of the available maps.

The final maps were integrated into the CMAP comparative map viewer tool http://gmod.org/wiki/CMap in MoccaDB and displayed using CIRCOS [28].

### BAC identification and sequencing

The BAC Clone 111O18 was identified in the *C. canephora *BAC library using high-density filter hybridizations and PCR amplifications with two coffee genes (SGN-U311255 and SGN-U628122) corresponding to genes coded BAC19.5 and BAC19.3 present at the ovate locus (AF273333). BAC sequencing was performed using the Sanger method (GATC) with a final quality of Phred 20 (Accession#: HM635075).

### Sequence analysis and annotation methods

The final BAC sequence was analyzed and annotated similarly as in Guyot et al. [23]; and Yu et al. [29]. De novo prediction of TEs was performed manually and putative TEs described as in previous studies [23,30].

### Analysis of the microsynteny with solanaceae and *Vitis *genomes

Local conservation of gene order and orientation was investigated between the coffee tree and Solanaceae by direct comparison of orthologous BAC sequences downloaded from NCBI: EF517793 petunia, EF517791 eggplant, EF517792 pepper, AF273333 tomato and EF517794 potato. Sequence comparisons were computed by Dotter and BLAST. The orthologous grapevine ovate region was identified by BLAST searches using predicted coffee coding regions as queries similarly as in Guyot et al. (2009) and Yu and Guyot et al. (2011) [23,29].

## Results

### Macrosynteny between the coffee tree and the tomato using COSII mapping data

A set of 867 Conserved Ortholog Set II (COSII) loci was selected including 430 and 755 sequences anchored on coffee and tomato genetic maps, respectively. Some of them have recently been used to establish a high-resolution map in coffee [14]. In the present study, this map has been completed to end up with 467 markers covering a total distance of 1331 cM. Using the updated coffee and tomato genetic maps the coffee-tomato comparison was reassessed and shared 318 common markers (Additional file 1: Figure S1) leading to a total of 27 syntenic blocks (Additional file 2: Table S1). The mean number of COSII per syntenic block was six. The mean size of each syntenic block was 18.2 cM on the coffee map and 15.9 cM on the tomato map (Table 1). The highest number of syntenic relationships was established between coffee Linkage Group (LG) G and tomato LG 2 with 16 COSII orthologous relationships (over 50 cM for coffee and 63.5 cM for tomato).

**Table 1:** Number of orthologous markers and characteristics of conserved Syntenic segment between coffee, tomato and grapevine genomes

Comparative maps	# Orthologous markers	# CSS	Mean CSS size	Max CSS size	Mean number of syntenic marker per CSS	Mean distance between adjacent syntenic markers
Coffee *vs*. Tomato	318	27	18.2 cM 15.9 cM	50 cM 70.5 cM	6 (Max: 16)	4.3 cM 3.4 cM

Coffee *vs*. Grapevine	299	29	14.3 cM 4.4 Mb	46.25 cM 15.6 Mb	6 (Max: 14)	3 cM 0.9 Mb

Tomato *vs*. Grapevine	470	45	13.6 cM 3.9 Mb	69.6 cM 11.7 Mb	5 (Max: 17)	3 cM 0.9 Mb

In four cases, all the syntenic blocks defined on a single coffee LG were related to a single tomato LG: D and 3, H and 8, J and 4, K and 5 (Additional file 2: Table S1).

### Macrosynteny between the coffee tree and the grapevine by *in silico *comparative mapping of COSII sequences

Coffee and tomato unigene sequences for each mapped COSII locus were then used as queries to the BLAST against a total of 485 Mbp of the grapevine genome assembled and released to the public http://www.genoscope.cns.fr/externe/GenomeBrowser/Vitis/ to identify coffee-grapevine homologs. Out of a total of 430 COSII loci mapped in coffee, 356 (83%) were found conserved on available grapevine genomic sequences, while 74 were found absent using the BLASTN E-value < 10^-6 ^as a cutoff (See Material and Methods). Out of these 74 COSII sequences, apparently absent from the grapevine genome, 26 were found conserved in grapevine-expressed sequences and 69 were found conserved in grapevine annotated CDS (using the BLASTX algorithm, E-value < 10^-5^). These results suggest that most of the initially absent COSII were finally detected on the grapevine genome but with higher E-values.

Out of the 356 COSII sequences conserved between coffee and grapevine, 299 (84%) mapped to a single locus on the grapevine genome, and were then considered as putative orthologs of coffee COSII loci since COSII were initially selected to correspond to single copy genes [7]. These findings are consistent with an absence of recent polyploidy in the grapevine genome. However, 57 COSII loci gave multiple matches and may correspond to members of gene families specifically expanded in grapevine genomes compared to coffee or tomato ones, whose sequences were used for the COSII loci definition [7]. Consequently, as this makes the grapevine genome more difficult to identify and scrambling our interpretation of the results, these COSII loci were excluded.

Out of the 299 single-locus grapevine COSII sequences, 282 were found on assembled pseudochromosomes. Four putative orthologs fell into segments assigned to known chromosomes but with unknown positions and 13 orthologs were found in contigs unallocated to specific grapevine chromosomes (Additional file 3: Table S2). These 17 COSII sequences were then excluded from our analysis.

The distribution of COSII sequences conserved along coffee and grapevine chromosomes showed that the putative orthologs are distributed all along the coffee linkage groups. The 282 single-locus conserved COSII sequences were further used for the macrosynteny analysis.

**Figure 2:**
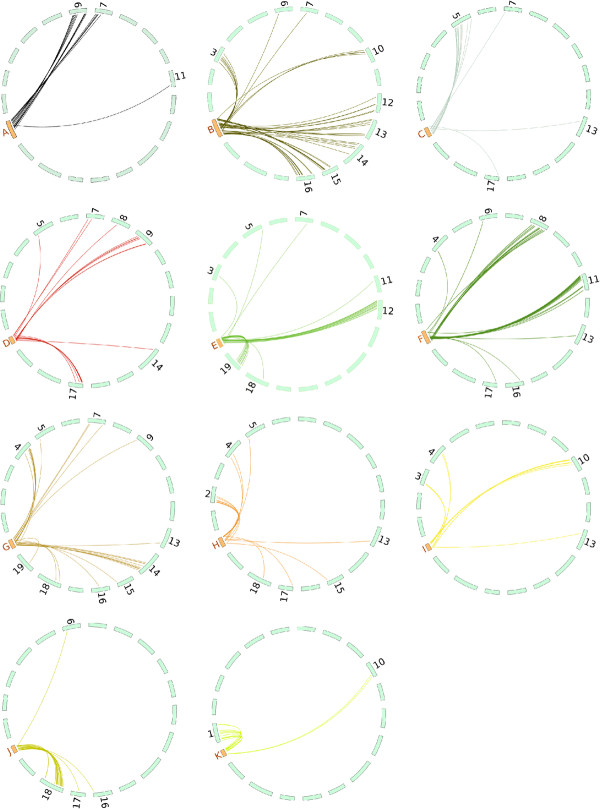
**Macrosyntenic relationships between each of the 11 coffee tree Linkage Groups and the 19 grapevine pseudo-chromosomes based on *in silico *mapping of mapped coffee COSII genes**. The coffee tree linkage groups (identified by letters) are represented in orange and the 19 grapevine pseudo-chromosomes are represented in blue. Each line links the position of a unique orthologous COSII gene between coffee linkage groups and grapevine chromosomes identified by BLAST searches as described in Materials and Methods.

Direct orthologous relationships between each of the 11 coffee Linkage Groups (LG) against the 19 pseudo-chromosomes of grapevine is displayed on Figure 2. These alignment results of COSII loci with single-locus putative orthologs in the grapevine were carefully analyzed to determine syntenic relationships. Despite differences between basic chromosome numbers and genome size between coffee and grapevine (respectively ~704 Mbp for 11 chromosomes [3] and ~475 Mbp for 19 chromosomes [16]), the analyses of the 282 single-locus COSII loci revealed that the synteny relationships are substantial but fractionated, through the conservation of numerous blocks. In total, the 282 conserved single-locus COSII sequences allowed to draw 29 syntenic blocks between the 11 coffee LGs and the 19 grapevine chromosomes (Additional file 4: Table S3). The mean number of syntenic COSII per blocks was 6 with a mean number of 2 interspaced non syntenic markers. The mean size of each syntenic block was 14.3 cM on the coffee map corresponding to 4.4 Mb in the grapevine genome (Table 1). The highest number of syntenic relationships was established between coffee LG E and the grapevine chromosome 12, with 14 COSII orthologous relationships (over 9.9 Mb for grapevine and 46 cM for coffee).

**Figure 3:**
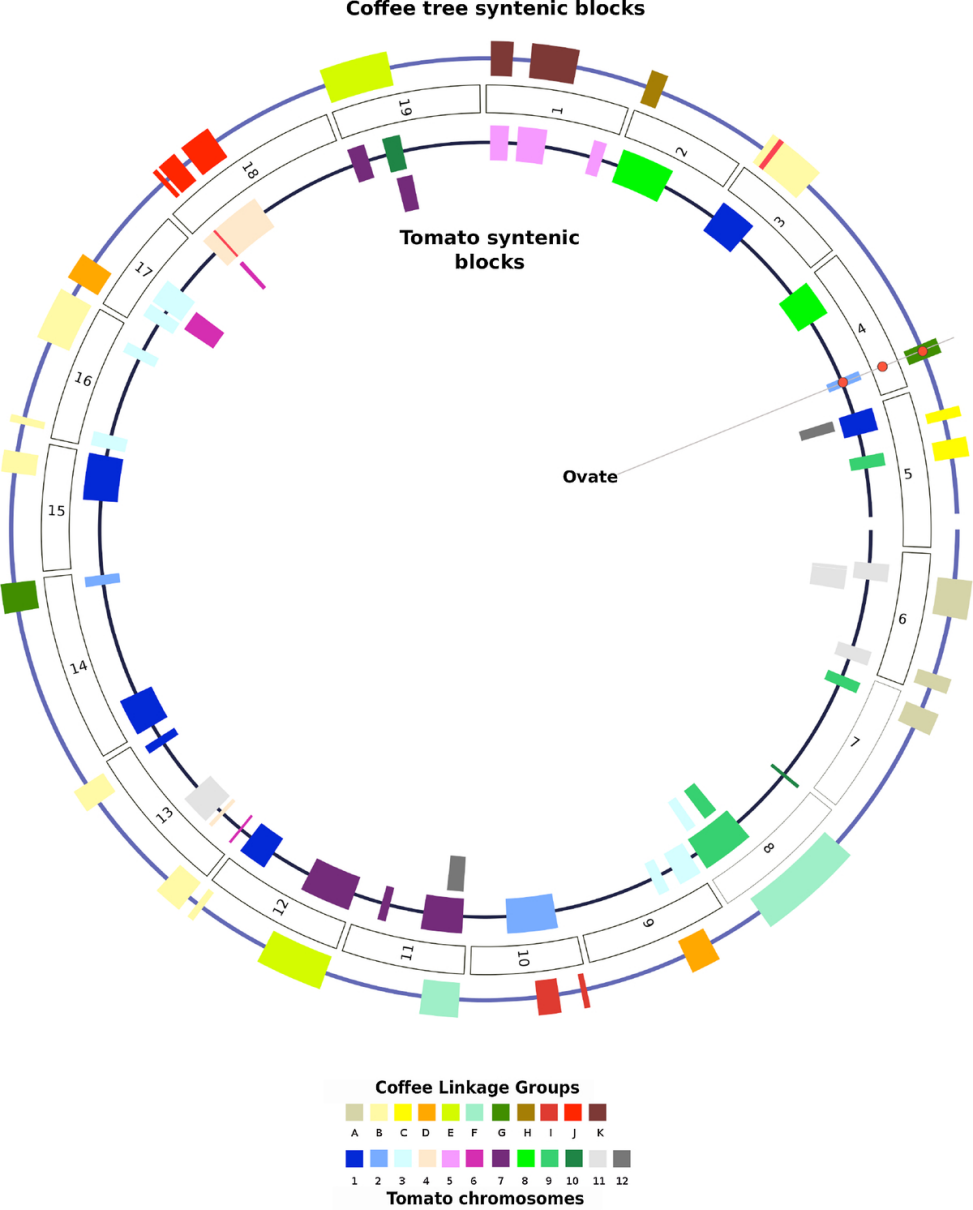
**Circle diagram of the syntenic relationships in the coffee tree and the tomato, relative to the grapevine pseudo-chromosomes**. The central circle represents the grapevine genome. Outside and inside circles represent syntenic blocks in coffee tree and tomato genomes, respectively. Syntenic blocks were described in Supporting information. For syntenic blocks, each color represents a different Linkage Group in coffee tree and in tomato genomes. Red dots represent the relative location of the *ovate *genes on the physical map of the grapevine genomes and on the genetic map of the coffee tree and the tomato.

In most of the cases, coffee LGs contain syntenic blocks that map to several grapevine chromosomes indicating that numerous rearrangements such as translocations and inversions have occurred since the separation of the two species. In the most extreme case i.e. coffee LG B, the longest linkage group, seven syntenic blocks were found conserved in five different grapevine chromosomes. In contrast, evidence is also given for significant conservation of coffee LGs with grapevine chromosomes: LG C and grapevine chromosome 5, LG J and chromosome 18 and LG K and chromosome 1 (Additional file 4: Table S3) suggesting that the chromosomal organization of these LGs might be ancestral to the Asterids and Rosids lineages (Figure 3).

### Macrosynteny between the tomato and the grapevine by *in silico *comparative mapping of COSII loci

Mapped tomato COSII sequences were used to establish the synteny with grapevine sequences as we did to establish the synteny between coffee and grapevine. Out of a total of 755 COSII loci mapped in the tomato, 567 (75%) were found conserved on the available grapevine genome, while 188 were not found. Out of the 567 COSII sequences conserved between tomato and grapevine, 470 (82.9%) mapped to a single locus on the grapevine genome and were thus considered as putative orthologs of tomato COSII loci. A total of 45 syntenic blocks were observed between the two species (Additional file 5: Table S4). The mean number of syntenic COSII per blocks was five with a mean number of four interspaced non syntenic markers. The mean size of each syntenic block was 13.6 cM on the tomato genetic map corresponding to 3.9 Mb in the grapevine genome (Table 1). The highest number of syntenic relationships was established between tomato LG 4 and the grapevine chromosome 18 (Additional file 5: Table S4), with 17 COSII orthologous relationships (over 11.7 Mb for grapevine and 69.6 cM for tomato). Unlike coffee, no conservation between specific tomato LG and grapevine chromosome was observed because tomato CSSs from a unique LG always mapped to several grapevine chromosomes.

### Macrosynteny among coffee, tomato and grapevine genomes

Based on the pairwise synteny established here, we drew an overview of the macrosyntenic relationships among coffee tree, tomato and grapevine genomes based on COSII-mapped loci (Figure 3). By comparing the location of these blocks among the three genomes, it is clear that numerous blocks that we identified in one pairwise analysis were found to completely or partially overlap blocks identified in other pairwise analyses, indicating that a substantial number of blocks of synteny may be conserved among the three species. All grapevine chromosomes were found covered by at least one conserved block of synteny from the two other species. Three examples of the detailed analysis of macrosynteny are shown in Additional file 6: Figure S2. Blocks of synteny appear differentially rearranged among coffee, tomato and grapevine, suggesting that profound reorganization of the genomes of the three species has occurred since their divergence. All the syntenic blocks found on the coffee Linkage Group C (LG C) are orthologous to grapevine chromosome 5 (V5) but combine two blocks with tomato Linkage Groups 1 and 9, suggesting that a translocation occurred in the tomato compared to coffee and grapevine. Thus, LG C and V5 may represent the ancestral chromosomal arrangement. Similarly, the coffee Linkage Group D displays only two adjacent syntenic blocks present on the tomato Linkage Group 3 while they spread on two grapevine chromosomes (9 and 17). In this case we may suppose that the grapevine experienced a chromosomal break followed by a translocation of the block after the separation of the Rosid and Asterid clades or a break in the shared lineage. The majority of the tomato Linkage Group 7 was found conserved with two segments in the coffee Linkage Groups E and F, suggesting a breakpoint arrangement in coffee as previously reported [14]. Each coffee chromosomal segment was found further fragmented into two syntenic fragments in grapevine chromosomes 19, 12, 8 and 11 (Additional file 6: Figure S2). These combined results suggest that the coffee tree, tomato and grapevine genomes, although presenting numerous conserved segments, have undergone extensive rearrangements leading to specific chromosomal evolution since their divergence from their last common ancestor.

### Synteny of the *ovate *regions among the coffee tree, tomato and grapevine species

To determine if agronomical important genes may be conserved in syntenic blocks between the coffee tree, the tomato and the grapevine, we decided to identify, map and compare the *ovate *regions in the coffee tree, the grapevine and several Solanaceae. The *ovate *locus was previously identified on tomato Linkage Group 2 in a major QTL affecting fruit shape [31]. It is also involved in determining fruit shape in pepper [32]. This region was sequenced in the tomato [33], in four other Solanaceae species [34] and in *Antirrhinum *[35].

**Figure 4:**
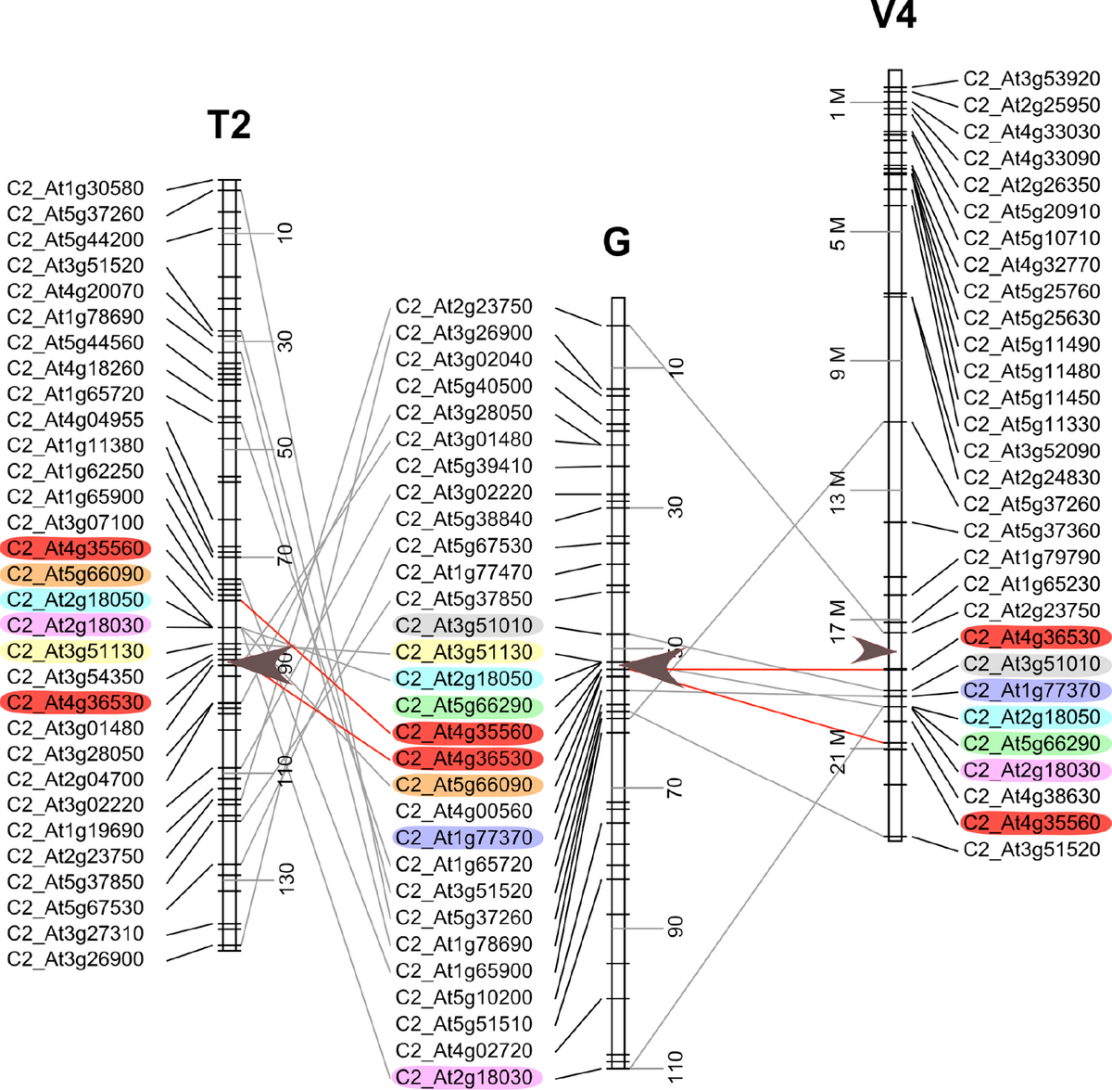
**Detailed synteny between coffee Linkage Group G, tomato Linkage Group 2 and grapevine chromosome 4**. Lines connect common COSII loci along coffee Linkage Group G, tomato Linkage Group 2 (T2) and grapevine chromosome 4 (V4). Genetic distances in cM are indicated for coffee and tomato Linkage Groups and physical distance in Mb are indicated for the grapevine chromosome. Colored COSII loci highlight conserved markers around *ovate *loci in coffee, tomato and grapevine chromosomes. Brown arrowheads indicate the relative positions of the *ovate *loci.

Using high-density filter hybridizations and PCR amplifications, we isolated the BAC Clone 111O18, from a *C. canephora *BAC library [36] carrying two orthologous single-copy genes, present in the tomato *ovate *region. This BAC clone was completely sequenced and analyzed (174 kb, accession # HM635075). Out of a total of 23 predicted coding regions identified along BAC sequences (Additional file 7: Table S5 and Additional file 8: Figure S3), Gene 6 (*g6*) encodes a putative protein with high similarities with the *ovate *protein from the tomato (AAN17752) [31]. The coffee BAC Clone 111O18 sequence was used to design microsatellite markers. Segregation analysis in the BP409 × Q121 cross progeny shows an unique locus on Linkage Group G (53 cM) [9]. The genetic position of the BAC Clone 111O18 is flanked by two COSII loci mapped (C2_At4g35560, LG G 53 cM and C2_At4g36530, LG G 53 cM). These two COSII loci are mapped and conserved within a syntenic block in tomato Linkage Group 2 and grapevine Chromosome 4 (Figure 4). Using Blastn we identified the orthologous segment corresponding to the *ovate *region in the genomic sequences of the grapevine. A segment of 234 kb (positions 17,954-17,168 Mb) on grapevine Chromosome 4 was finally identified to contain a gene similar to *ovate *from tomato (Figure 4).

To determine the relative position of the *ovate *region in the tomato we used Blast searches of COSII and *ovate *loci against the recent release of the preliminary tomato chromosome assembly. The ovate gene was identified on chromosome 2 (position 42,945 kb) between the C2_At4g36530 and C2_At3g01480 COSII genes (Figure 4). Our combined data suggest that the *ovate *region is conserved within a syntenic block between the coffee tree, the tomato and the grapevine.

### Microsynteny among solanaceae, the coffee tree and the grapevine at the ovate locus

**Figure 5:**
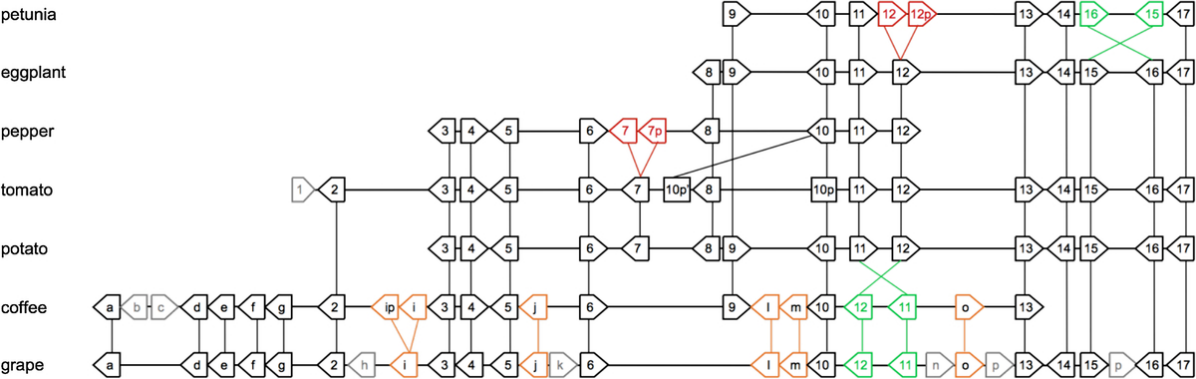
**Microsynteny in the *ovate *orthologous regions of coffee tree, grapevine and Five different species of Solanaceae**. The sequenced segments of the potato (*Solanum bulbocastanum*), tomato (*Solanum lycopersicum*), pepper (*Capsicum annuum*), eggplant (*Solanum melongena*) and petunia (*Petunia inflata*) [34] were compared to orthologous segments in the coffee tree (BAC clone 111O18) and the grapevine genome. Arrows symbolize the orientation of predicted coding regions. Letters and numbers linked to arrows indicate orthologous gene families as defined in Additional file 9: Table S6. Gene ovate belongs to the gene family 6. Lines link coding regions between conserved syntenic segments. Duplicate genes are represented in red while inverted genes are shown in green. Genes conserved between coffee and grape but absent in orthologous locations in the five *Solanaceae *species were indicated in orange. Genes present in only one species are indicated in grey.

As a complement to the macrosynteny study and to determine if conservation of the genome macrostructure identified by COSII markers might be accompanied by microstructure conservation, we compared genomic sequences between the coffee tree, several Solanaceae and the grapevine at the *ovate *regions. Recently, orthologous BAC clones to the tomato *ovate *locus from petunia (*Petunia inflata*), eggplant (*Solanum melongena*), pepper (*Capsicum annuum*) and a wild potato (*Solanum bulbocastanum*) were identified, sequenced and compared [34]. Here, genome microstructure conservation studies were first conducted between orthologous BAC sequences of the coffee tree and Solanaceae. Pairwise comparisons, using the program dotter [37], revealed that nucleotide conservation was strictly limited to the exons. The overall gene content and order was found conserved. A total of 10 genes were found conserved, of which eight share identical order and orientation. Interestingly, the *ovate *gene (Putative orthologous gene family 6, Figure 5) was found conserved at an orthologous location. However, local missing or extra genes and a local gene inversion created small differences between coffee and five Solanaceae species (Figure 5 and Additional file 8: Figure S3). For example putative orthologous genes 7 and 8 were present in Solanaceae (respectively in three and four species, covered by the sequenced BACs) but not in the coffee tree, while six genes in the coffee tree were not found in the Solanaceae BAC sequences that covered orthologous positions of these genes (Figure 5). In addition to gene losses, two genes have apparently undergone local inversion, resulting in differences in gene order and orientation in the coffee tree compared to all other Solanaceae species. Despite these small differences, the comparison between coffee and Solanaceae at the *ovate *locus reveals strong overall conservation. Conservation between the coffee and the grapevine *ovate *regions (grapevine chromosome 4, positions 17,954-17,168 Mb) were also investigated. Surprisingly, the level of conservation appears fairly high between coffee and grapevine, since a total of 20 coffee genes encounter putative orthologs along the segment on grapevine chromosome 4 (Figure 5). Beside the gene content, gene order and orientation appears strictly conserved. Only three extra-predicted genes in coffee and four in grapevine perturb the high level of synteny observed. Very few type II transposable elements were detected in this region (Additional file 7: Table S5 and Additional file 8 Figure S3). None of these elements, found in one plant, has homologues in the other two families. The revealed synteny and gene order is not disturbed by these elements.

In conclusion, the overall conservation analysis of the gene content, order and structure among five species in Solanaceae, the coffee tree and the grapevine suggests that microstructure may be conserved in syntenic blocks among coffee, tomato and grapevine genomes. Surprisingly our analysis indicates better conservation of the microsynteny between the coffee tree and the grapevine than between the coffee tree and the Solanaceae at the *ovate *locus. The detailed analyses of the syntenic segment where Solanaceae, coffee tree and grapevine sequences are overlapping (as defined by the arrow on Figure 5) show complete syntenic conservation of eight genes between the coffee tree and *Solanaceae *(conservation of order and orientation) while two other syntenic genes have undergone an inversion, representing the only major exception to synteny. Here eight extra genes were not found conserved between these two species. While, analyses of the same syntenic segment between coffee and grapevine show the complete conservation of 12 predicted genes in the same order and orientation, only one gene has undergone inversion (*g4*, Figure 5). Five extra predicted genes were not found conserved between compared segments (Figure 5). These analyses suggest more robust syntenic relationships between the coffee tree and the grapevine than between the coffee tree and Solanaceae at the *ovate *locus, despite a greater evolutionary distance between coffee and grapevine than between coffee and Solanaceae (Figure 1).

## Discussion

### Genetic/physical comparative mapping identifies some degree of macrosynteny among distantly-related species

Macrosynteny generally refers to conserving the linkage of genetic loci among different species. Macrosynteny may be revealed either by comparative genetic mapping of markers or, more recently, by physical mapping of conserved sequences within sequenced reference genomes [38,39]. Establishing the syntenic relationships among different species offers a valuable tool for understanding their chromosomal evolution and facilitates the transfer of genetic and genome information from a model genome to the species under investigation [40]. This concept has been successfully developed and applied in different plant families such as Solanaceae, Brasicaceae and Poaceae [40]. In grass species, despite the enormous difference in chromosome number and genome size, some genes of agronomic interest were found conserved in shared syntenic blocks between relatively distant species [41,42]. So far, however, comparative mapping studies have shown that shared macrosynteny has been conserved among species of the same family. More generally, it appears that synteny relationships are more often conserved among closely related species and decline with the increase of phylogenetic distances. In dicotyledonous plants, comparative mapping studies demonstrated the complexity of establishing shared macrosynteny among distant species [19,43]. The main problems are due to difficulties in uncovering orthologous markers that may be transferable across distant families, the level of genome rearrangements increasing according to the phylogenetic distance between species, and ancestral whole genome duplication that may scramble syntenic relationships. Using bioinformatic approaches, a large set of single-copy orthologous genes (COSII) shared between Asterid plant species (including *C. canephora *and *S. lycopersicum*) and the model Rosid plant genome *A. thaliana *has been identified [7] and used for comparative genetic mapping and chromosomal evolution studies in the Solanaceae [10-13]. Thanks to the intrinsic feature of tagging orthologous single loci, Lefebvre-Pautigny et al. (2009) [14] further demonstrated that COSII loci can be used for comparative mapping across Rubiaceae (coffee tree) and Solanaceae (tomato, potato...) plant families and that shared synteny was detected among these species. In order to investigate chromosome evolution over large phylogenetic distances and to decipher chromosome evolution between Solanaceae and Rubiaceae with more precision, we assessed the relationships of coffee tree and tomato genomes with grapevine by *in silico *mapping approaches of genetically mapped COSII sequences. Pairwise comparisons between tomato/grapevine and coffee tree/grapevine brought to light an unsuspected level of shared synteny with the grapevine genome, considering the phylogenetic distances between the Rubiaceae and the Solanaceae on one hand, and between the Rubiaceae and the Vitaceae on the other. These families belong to different clades: Asterids for the Solanaceae and the Rubiaceae and Rosids for the Vitaceae. Both comparisons displayed synteny fragmented into relatively small blocks: 4.4 Mb and 3.9 Mb in average, when grapevine is compared to coffee and tomato, respectively. Several large blocks of shared synteny were also detected covering up to 15 Mb in the grapevine genome or containing up to 14 COSII syntenic markers between coffee and grapevine. Beside phylogenetic distances, this level of conservation is also particularly striking considering the large chromosome number and genome size differences between grapevine (n = 19, 475 Mb) compared to both tomato (n = 12; 965 Mb) and coffee tree (n = 11; 704 Mb). Our *in silico *mapping analysis supports the absence of recent duplication in the grapevine genome. Whole genome duplications have deep consequences on genome organization. It may promote a more complex genome structure through a high level of chromosome rearrangements, making it difficult to clearly identify syntenic blocks. In previous comparative mapping studies between dicotyledonous species, such as Arabidopsis, potato, sugar beet, sunflower and *Prunus*, ancestral and conserved syntenic segments were discovered using genetic map based on ESTs [22]. However the duplicated nature of the referential Arabidopsis genome gave a complex picture of the shared macrosynteny [22,43]. In contrast, the absence of paleo-polyploidy events ever since the divergence of the tomato, coffee and grapevine genomes may explain this particularly stable conservation of the ancestral genome structure, which made it easier to detect ancestral fragments of shared macrosynteny.

Several grapevine chromosomes showed a low level or complete absence of shared synteny when compared to coffee and tomato. The same observation is true with several parts of the coffee LGs that are covered with a low COSII loci number. In this case, the increase of the number of COSII loci, particularly in regions that are less covered, will improve the resolution in detecting the shared synteny between coffee and grapevine. In the near future, the fullysequenced genomes of grapevine, tomato and the forthcoming sequence of the coffee genome promise improvements in detecting synteny, particularly in chromosomal segments which are not covered.

### Microsynteny can be conserved in ancestral shared macrosyntenic blocks among tomato, coffee tree and grapevine genomes

In contrast with macrosynteny, significant microsynteny was previously well established between distantly-related dicotyledonous species [44,45], and recently microsynteny were even detected between the coffee tree and different reference genomes such as the grapevine [23]. However most of the interspecies analysis of the microsynteny reported so far were established without information on macrostructure conservation or with evidence of lack of macrosynteny conservation [45].

Here, we compared the level of microsynteny in conserved ancestral macrosyntenic blocks at the *ovate *orthologous loci between grapevine, coffee tree and five Solanaceae species. The *ovate *locus intervenes in the control of the fruit shape development and has been identified in the tomato, on a BAC clone that has been completely sequenced [31,33]. Moreover, orthologous BAC clones from four other Solanaceae (potato, eggplant, pepper and petunia) were identified, sequenced and compared at the gene level [34]. A high degree of collinearity was observed between these species that separated from a common ancestor about 27-36 million years--and generations--ago. Using sequence information from the tomato *ovate *locus we characterized the orthologous segments in the coffee tree and the grapevine and we showed that these orthologous segments belong to ancestral syntenic blocks between tomato Linkage Group 2, coffee Linkage Group G and grapevine chromosome 4. At the gene level, the level of conservation was particularly high considering the relative phylogenetic distances between the species studied. Yet, several isolated disruptions of conservation were noted, such as gene loss, local gene inversion and tandem gene duplication (Figure 5), but similar to previous observations of microsynteny in plants [45,46]. A remarkable point emerges in our study with a stronger-than-expected level of conservation observed between coffee and grapevine segments. An unexpectedly high level of microsynteny between *Fragaria *and grapevine compared to a low level of synteny between *Fragaria *and *Arabidopsis *was also observed [19], but this comparison only involved species, although distant, from the Rosid clade. Considering the phylogenetic distance between Solanaceae and Rubiaceae (Asterids) from the Vitaceae family (basal Rosid), we expected a stronger conservation of local microstructure within Solanaceae species and then, between Solanaceae and Rubiaceae. However, the microstructure here is clearly conserved more completely between coffee and grapevine segments than between coffee and the five Solanaceae species analyzed. Five different particular genes were found conserved between coffee and grapevine but completely absent in Solanaceae. Furthermore, an inversion of the transcriptional orientation of two genes was shared in all the Solanaceae segments in relation to the coffee or the grapevine counterparts. All these observations suggest that most of the rearrangements at the *ovate *locus occurred independently in the Solanaceae and most likely shortly after their divergence with the Rubiaceae family.

To date it is not clear why stronger conservation of microstructure was found between species that are separated by 114-125 million years of independent evolution and whether this conservation can be extended to other loci in coffee and grapevine genomes. However, it is interesting to note that grapevine and coffee trees are both woody plants with a long generational cycle of about four years, while the tomato and the Solanaceae considered in this study are all annual plants. Yet, in terms of generations, coffee/grapevine separation only took place about 28 and 31 million generations ago and this might be one of the reasons of the apparent higher conservation between them. This number is to be compared with the 83-89 million years that separate coffee trees and tomatoes and the same number of generations with respect to the tomato. Although no convincing comparative mapping studies have been conducted to date to test life-history impact on the genome structure, it has been demonstrated that rates of molecular evolution are consistently low in trees and shrubs, with relatively long generation times, as compared with related herbaceous plants, which generally have shorter generation times [47-49]. A comparative genomic sequence analysis of strawberry (*Fragaria*), a non ligneous but perennial plant, with other rosids [19] revealed also a high level of synteny with *Populus *and *Vitis*, two ligneous species, and an apparent stability of their genomes. Very few studies compared syntenic relationships between annual and perennial plants, a comparison between *Lolium perenne *(perennial ryegrass) and other Poaceae showed a good conservation of synteny and collinearity [50], but this study concerned herbaceous and monocotyledonous plants within a unique family. Comparative studies between annual and perennial dicotyledonous plants should be conducted to confirm or invalidate our hypothesis about the genome structure conservation among perennial vs. annual plant species.

Large-scale sequence data has enabled the detection of micro-collinear regions in less closely related species without apparent associated macrosynteny [45,51,52]. In our study, one of the few that compare distantly related species from the asterid and rosid clades at the genome organization level, we revealed unexpected genome macro-and microstructure conservation.

## Conclusion

We have used a large set of genetically mapped COSII loci on coffee tree and tomato and the public release of the grapevine genome to evaluate the synteny relationships between these distantly related genomes from species of agronomic interest. It appears that significant synteny can be detected through numerous small blocks, allowing a first analysis about the divergent chromosomal histories between tomato, coffee trees and grapevine. In addition, based on the observation of the *ovate *locus, syntenic blocks appear to be associated with an extensive conservation of the microstructure. However, Solanaceae microstructures appear much more different that the conservation between coffee tree and grapevine, suggesting a divergent and specific evolution of the locus in the Solanaceae prior to the separation with the Rubiaceae and that time factor alone does not explain the divergences. From an applied perspective, the detailed analysis of blocks of synteny over these distantly related plant species is certainly of great interest in comparative genomic approaches to identified genes of interest.

## Abbreviations

BAC: bacterial artificial chromosome; BLAST: basic local alignment search tool; CSS: conserved syntenic segment; EST: expressed sequence tag; LG: linkage groups; PCR: polymerase chain reaction; RFLP: Restriction fragment length polymorphism; SNP: single nucleotide polymorphism; SSR: simple sequence repeats.

## Competing interests

The authors declare that they have no competing interests.

## Authors' contributions

FL-P provided coffee tree mapping information for comparative mapping. MR and FL-P designed coffee COSII markers. RG, CTD and VP carried out the COS analysis and wrote associated sections of the manuscript. RG, FL-P and CTD constructed the linkage maps. CTD extended MoccaDB and input data into the database. RG and VP assisted in the extension and designing of the database. SH and AdK coordinated the sequencing and microsynteny work. MR, FL-P and DC coordinated the genetic mapping work. TL provided the BAC clone and with DC, and MR contributed to its sequencing. PH, SH, AdK, and DC coordinated research activities between the laboratories. DC and AdK coordinated the project, conceived and designed the experiments. All authors read and approved the final manuscript.

## Supplementary Material

Additional file 1 **Figure S1 Macrosyntenic Relationships between each of the 11 Coffee Linkage Groups and the 12 Tomato Linkage Groups based on Mapped Coffee COSII Loci**. Click here for file

Additional file 2 **Table S1 Syntenic Blocks between Coffee and Tomato Linkage Groups**. Click here for file

Additional file 3 **Table S2 Distribution of the Coffee COSII Sequences Mapped on Grapevine Pseudo-Chromosomes**. Click here for file

Additional file 4 **Table S3 Syntenic Blocks between Coffee Tree Linkage Groups and the Pseudo-Chromosomes of the Grapevine Genome**. Click here for file

Additional file 5 **Table S4 Syntenic Blocks between Tomato Chromosomes and the Pseudo-Chromosomes of the Grapevine Genome**. Click here for file

Additional file 6 **Figure S2 Detailed Examples of Macrosynteny between the Coffee Tree, the Tomato and the Grapevine**. Click here for file

Additional file 7 **Table S5 List of Identified Genes in the Coffee Tree (*C. canephora*) BAC clone 111O18.** Click here for file

Additional file 8 Physical map and annotation of the 174,135 bp of the C. canephora BAC clone 111O18. Click here for file

Additional file 9 Table S6 List of orthologous predicted genes in the ovate regions of *C. canephora*, Solanaceae and the grapevine genome. Click here for file
