# Supplementary material for: Ancestral synteny shared between distantly-related plant species from the asterid (Coffea canephora and Solanum Sp.) and rosid (Vitis vinifera) clades
Source: BMC Genomics. 2012 Mar 20;13:103. doi: 10.1186/1471-2164-13-103 (PMC3372433; doi:10.1186/1471-2164-13-103)
Supplement: Additional file 1 — Figure S1 Macrosyntenic Relationships between each of the 11 Coffee Linkage Groups and the 12 Tomato Linkage Groups based on Mapped Coffee COSII Loci. [file 1471-2164-13-103-S1.DOC]

**Supporting Information** Guyot *et al*., “Ancestral Synteny Shared between Distantly-Related Plant Species from the Asterid (*Coffea canephora* and *Solanum* sp.) and Rosid (*Vitis vinifera*) Clades”


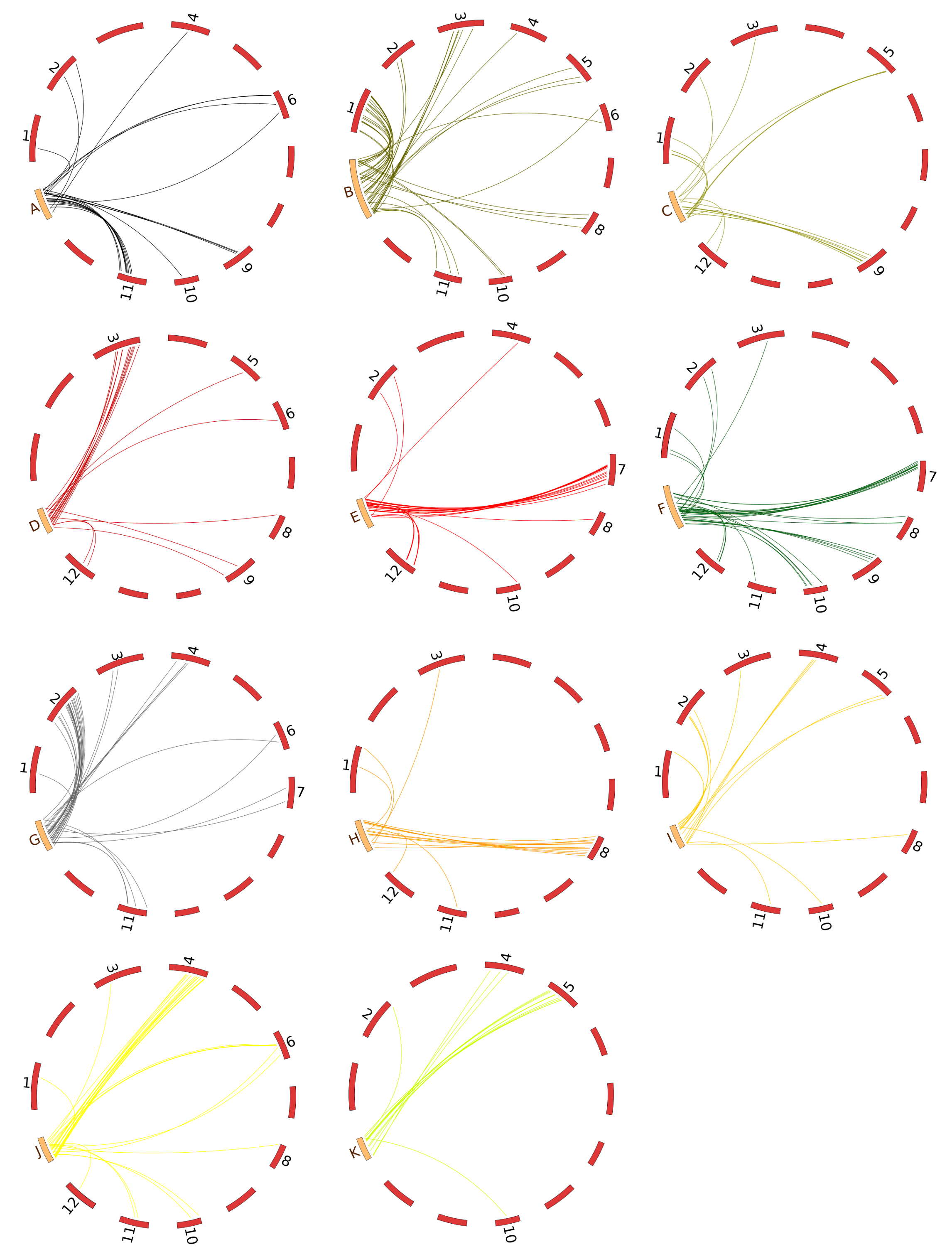


Fig S1. Macrosyntenic relationships between each of the 11 coffee Linkage Groups and the 12 tomato Linkage Groups based on mapped coffee COSII loci. The coffee linkage groups (identified by letters) are represented in orange and the 12 tomato Linkage Groups are represented in red. Each line links the position of unique orthologous COSII loci between coffee and tomato linkage groups as described in Material and Methods.
