# Supplementary material for: Ancestral synteny shared between distantly-related plant species from the asterid (Coffea canephora and Solanum Sp.) and rosid (Vitis vinifera) clades
Source: BMC Genomics. 2012 Mar 20;13:103. doi: 10.1186/1471-2164-13-103 (PMC3372433; doi:10.1186/1471-2164-13-103)
Supplement: Additional file 2 — Table S1 Syntenic Blocks between Coffee and Tomato Linkage Groups. [file 1471-2164-13-103-S2.DOC]

**Supporting Information** Guyot *et al*., “Ancestral Synteny Shared between Distantly-Related Plant Species from the Asterid (*Coffea canephora* and *Solanum* sp.) and Rosid (*Vitis vinifera*) Clades”

| **CSS #** | **Coffee LG** | **Coffee CSS position (cM)** | **Coffee CSS position (cM)** | **Coffee CSS size (cM)** | **COS** | **COS** | **Tomato LG** | **COS** | **COS** | **Tomato CSS position (cM)** | **Tomato CSS position (cM)** | **Tomato CSS size (cM)** | **Coffee COSII per block** | **Coffee COSII synthenic per CSS** |
| --- | --- | --- | --- | --- | --- | --- | --- | --- | --- | --- | --- | --- | --- | --- |
| 1 | A | 35 | 69 | 34 | C2_At5g04590 | C2_At5g60600 | 11 | C2_At5g04590 | C2_At5g59960 | 56 | 79 | 23 | 11 | 8 |
| 2 | A | 70 | 92 | 22 | C2_At2g28490 | C2_At5g58490 | 11 | C2_At5g58490 | C2_At5g59960 | 97 | 103 | 6 | 8 | 3 |
| 3 | A | 90 | 106 | 16 | C2_At5g58410 | C2_At5g43940 | 9 | C2_At5g43940 | C2_At4g02680 | 47 | 56 | 9 | 9 | 5 |
| 4 | B | 0 | 19 | 19 | C2_At3g02910 | C2_At5g62390 | 3 | C2_At3g02910 | C2_At3g49260 | 54 | 76 | 22 | 15 | 7 |
| 5 | B | 87 | 95 | 7 | C2_At1g49510 | C2_At2g14910 | 1 | C2_At3g19630 | C2_At2g14910 | 146 | 151 | 4 | 7 | 5 |
| 6 | B | 130 | 145 | 14 | C2_At1g64850 | C2_At3g63490 | 1 | C2_At3g63490 | C2_At3g20230 | 44 | 48 | 4 | 4 | 4 |
| 7 | B | 150 | 166 | 16 | C2_At2g38730 | C2_At1g54520 | 1 | C2_At2g38730 | C2_At1g54520 | 93 | 101 | 9 | 6 | 3 |
| 8 | B | 205 | 223 | 17 | C2_At1g48050 | C2_At2g45620 | 1 | C2_At1g48050 | C2_At2g45620 | 58 | 69 | 11 | 8 | 4 |
| 9 | C | 0 | 18 | 18 | C2_At3g06790 | C2_At3g23590 | 1 | C2_At3g06790 | C2_At4g15520 | 32 | 47 | 15 | 11 | 5 |
| 10 | C | 22 | 50 | 28 | C2_At1g04190 | C2_At3g16840 | 9 | C2_At1g05385 | C2_At3g24050 | 65 | 97 | 32 | 6 | 6 |
| 11 | D | 20 | 41 | 21 | C2_At1g80360 | C2_At1g68660 | 3 | C2_At1g52590 | C2_At1g80360 | 128 | 150 | 21 | 11 | 6 |
| 12 | D | 49 | 57 | 7 | C2_At5g41040 | C2_At3g48610 | 3 | C2_At5g41040 | C2_At5g63460 | 76 | 76 | 0 | 6 | 4 |
| 13 | E | 56 | 78 | 22 | C2_At4g03210 | C2_At2g38020 | 7 | C2_At2g20690 | C2_At2g38020 | 42 | 49 | 7 | 13 | 8 |
| 14 | E | 67 | 78 | 11 | C2_At2g42620 | C2_At1g06550 | 12 | C2_At2g42620 | C2_At4g11120 | 46 | 48 | 2 | 7 | 4 |
| 15 | F | 17 | 29 | 12 | C2_At2g37240 | C2_At5g06130 | 9 | C2_At2g37240 | C2_At5g06130 | 24 | 38 | 14 | 5 | 5 |
| 16 | F | 32 | 75 | 43 | C2_At5g34850 | C2_At5g58200 | 7 | C2_At5g20350 | C2_At3g26060 | 0 | 28 | 28 | 27 | 14 |
| 17 | F | 54 | 70 | 16 | C2_At2g06530 | C2_At1g19130 | 12 | C2_At2g06530 | C2_At2g06005 | 33 | 34 | 1 | 8 | 3 |
| 18 | G | 4 | 54 | 50 | C2_At2g23750 | C2_At5g66090 | 2 | C2_At4g35560 | C2_At3g26900 | 79 | 142 | 64 | 33 | 16 |
| 19 | G | 58 | 60 | 1 | C2_At1g65720 | C2_At1g78690 | 2 | C2_At3g51520 | C2_At1g65720 | 28 | 45 | 17 | 3 | 3 |
| 20 | G | 73 | 81 | 8 | C2_At1g34370 | C2_At3g62940 | 4 | C2_At3g62940 | C2_At4g39450 | 56 | 64 | 8 | 5 | 3 |
| 21 | H | 58 | 71 | 13 | C2_At5g20910 | C2_At5g25940 | 8 | C2_At5g25940 | C2_At2g24830 | 23 | 34 | 11 | 5 | 3 |
| 22 | H | 100 | 121 | 21 | C2_At1g62780 | C2_At1g32900 | 8 | C2_At1g62780 | C2_At1g32900 | 63 | 87 | 24 | 10 | 6 |
| 23 | I | 4 | 24 | 20 | C2_At3g52155 | C2_At1g65230 | 4 | C2_At3g52155 | C2_At1g65230 | 51 | 63 | 13 | 7 | 3 |
| 24 | I | 58 | 63 | 5 | C2_At4g21120 | C2_At1g29950 | 2 | C2_At5g46250 | C2_At1g29950 | 39 | 44 | 5 | 3 | 3 |
| 25 | I | 74 | 83 | 9 | C2_At2g16920 | C2_At4g35250 | 1 | C2_At2g16920 | C2_At4g35250 | 165 | 165 | 0 | 4 | 3 |
| 26 | J | 3 | 40 | 38 | C2_At1g63610 | C2_At1g78230 | 4 | C2_At1g35720 | C2_At1g63610 | 62 | 133 | 71 | 22 | 15 |
| 27 | K | 65 | 66 | 2 | C2_At1g14270 | C2_At1g70160 | 5 | C2_At1g70160 | C2_At1g14270 | 11 | 19 | 8 | 4 | 3 |

Table S1. Syntenic blocks between coffee and tomato Linkage Groups.
