# Supplementary material for: Ancestral synteny shared between distantly-related plant species from the asterid (Coffea canephora and Solanum Sp.) and rosid (Vitis vinifera) clades
Source: BMC Genomics. 2012 Mar 20;13:103. doi: 10.1186/1471-2164-13-103 (PMC3372433; doi:10.1186/1471-2164-13-103)
Supplement: Additional file 3 — Table S2 Distribution of the Coffee COSII Sequences Mapped on Grapevine Pseudo-Chromosomes. [file 1471-2164-13-103-S3.DOC]

**Supporting Information** Guyot *et al*., “Ancestral Synteny Shared between Distantly-Related Plant Species from the Asterid (*Coffea canephora* and *Solanum* sp.) and Rosid (*Vitis vinifera*) Clades”

|  | **Coffee linkage groups** | | | | | | | | | | |  |
| --- | --- | --- | --- | --- | --- | --- | --- | --- | --- | --- | --- | --- |
| **Vitis chr.** | **A** | **B** | **C** | **D** | **E** | **F** | **G** | **H** | **I** | **J** | **K** | **Total** |
| **1** |  |  |  |  |  |  |  |  |  |  | **15** | **15** |
| **2** |  |  |  |  |  |  |  | **8** |  |  |  | **8** |
| **3** |  | **7** |  |  | **1** |  |  |  | **3** |  |  | **11** |
| **4** |  |  |  |  |  | **1** | **11** | **2** | **2** |  |  | **16** |
| **5** |  |  | **14** | **1** | **1** |  | **1** | **1** |  |  |  | **18** |
| **6** | **18** | **1** |  |  |  | **1** |  |  |  | **1** |  | **21** |
| **7** | **5** | **1** | **1** | **2** | **1** |  | **3** |  |  |  |  | **13** |
| (7 random) |  |  |  |  |  |  | 1 |  |  |  |  | 1 |
| **8** |  |  |  | **1** |  | **10** |  |  |  |  |  | **11** |
| **9** |  |  |  | **6** |  |  | **1** |  |  |  |  | **7** |
| **10** |  | **4** |  |  |  |  |  |  | **6** |  | **2** | **12** |
| **11** | **1** |  |  |  | **1** | **17** |  |  |  |  |  | **19** |
| **12** |  | **6** |  |  | **15** |  |  |  |  |  |  | **21** |
| **13** |  | **8** | **1** |  |  | **1** | **1** | **1** | **1** |  |  | **13** |
| (13 random) |  |  |  |  |  | 1 |  |  |  |  |  | 1 |
| **14** |  | **6** |  | **1** |  |  | **11** |  |  |  |  | **18** |
| **15** |  | **8** |  |  |  |  | **1** | **1** |  |  |  | **10** |
| **16** |  | **12** |  |  |  | **1** | **1** |  |  | **1** |  | **15** |
| **17** |  |  | **1** | **9** |  | **1** |  | **1** |  | **1** |  | **13** |
| **18** |  |  |  |  | **1** |  | **2** | **1** |  | **25** |  | **29** |
| (18 random) |  |  |  |  |  |  |  |  |  | 2 |  | 2 |
| **19** |  |  |  |  | **11** |  | **1** |  |  |  |  | **12** |
| Unknown |  | 2 |  | 1 | 1 | 3 | 2 |  | 4 |  |  | 13 |
| **mapped** | **24** | **55** | **17** | **21** | **32** | **36** | **36** | **15** | **16** | **30** | **17** | **299** |
| *unmapped* | *11* | *19* | *9* | *10* | *12* | *18* | *20* | *10* | *5* | *8* | *9* | *131* |
| **Total** | **35** | **74** | **26** | **31** | **44** | **54** | **56** | **25** | **21** | **38** | **26** | **430** |

Table S2. Distribution of the coffee COSII sequences mapped on grapevine pseudo-chromosomes. The Coffee COSII marker sequences were used as a query to BLAST against the grapevine genomic sequences with a cut-off of the e-value of 10^-6^. Mapped Single-locus COSII sequences between coffee and grapevine and corresponding to 299 sequences are indicated. On these 299 single-locus grapevine COSII sequences, 282 were found on assembled “pseudo-chromosomes” whereas 4 putative orthologs fell into segments assigned to known chromosomes but with unknown positions (7 random, 13 random and 18 random); 13 orthologs were found in contigs that are unallocated to specific chromosomes.
