# Supplementary material for: Ancestral synteny shared between distantly-related plant species from the asterid (Coffea canephora and Solanum Sp.) and rosid (Vitis vinifera) clades
Source: BMC Genomics. 2012 Mar 20;13:103. doi: 10.1186/1471-2164-13-103 (PMC3372433; doi:10.1186/1471-2164-13-103)
Supplement: Additional file 4 — Table S3 Syntenic Blocks between Coffee Tree Linkage Groups and the Pseudo-Chromosomes of the Grapevine Genome. [file 1471-2164-13-103-S4.DOC]

**Supporting Information** Guyot *et al*., “Ancestral Synteny Shared between Distantly-Related Plant Species from the Asterid (Coffea canephora and Solanum sp.) and Rosid (Vitis vinifera) Clades”

| **CSS #** | **Coffee LG** | **Coffee CSS position (cM)** | **Coffee CSS position (cM)** | **Coffee CSS size (cM)** | **COS** | **COS** | **Vitis pseudochr.** | **COS** | **COS** | **Vitis CSS position (bp)** | **Vitis CSS position (bp)** | **Vitis CSS size (bp)** | **Coffee COSII per block** | **Coffee COSII synthenic per CSS** |
| --- | --- | --- | --- | --- | --- | --- | --- | --- | --- | --- | --- | --- | --- | --- |
| 1 | A | 47 | 63 | 16 | C2_At3g44600 | C2_At3g44880 | vitis-6 | C2_At3g44880 | C2_At2g27450 | 18 321 926 | 20 568 364 | 2 246 438 | 7 | 6 |
| 2 | A | 68 | 93 | 25 | C2_At5g60540 | C2_At5g58240 | vitis-6 | C2_At5g60540 | C2_At4g10360 | 3 629 899 | 9 375 962 | 5 746 063 | 12 | 10 |
| 3 | A | 101 | 111 | 11 | C2_At1g03150 | C2_At1g02475 | vitis-7 | C2_At1g03150 | C2_At1g02475 | 1 220 280 | 4 527 037 | 3 306 757 | 6 | 4 |
| 4 | B | 0 | 4 | 4 | C2_At3g02910 | C2_At3g03100 | vitis-16 | C2_At3g02910 | C2_At3g03100 | 1 143 709 | 2 254 869 | 1 111 160 | 5 | 3 |
| 5 | B | 7 | 22 | 16 | C2_At5g24170 | C2_At3g22660 | vitis-16 | C2_At3g22660 | C2_At3g49260 | 14 002 985 | 21 855 316 | 7 852 331 | 12 | 8 |
| 6 | B | 23 | 34 | 11 | C2_At3g56460 | C2_At3g55800 | vitis-13 | C2_At3g56460 | C2_At3g55800 | 112 594 | 4 389 672 | 4 277 078 | 4 | 3 |
| 7 | B | 83 | 96 | 13 | C2_At2g21290 | C2_At2g14260 | vitis-3 | C2_At2g21290 | C2_At2g14260 | 624 055 | 9 431 232 | 8 807 177 | 9 | 6 |
| 8 | B | 109 | 117 | 8 | C2_At1g10240 | C2_At2g02500 | vitis-12 | C2_At1g10240 | C2_At2g02500 | 21 229 307 | 22 269 518 | 1 040 211 | 3 | 3 |
| 9 | B | 143 | 150 | 6 | C2_At5g26880 | C2_At2g38730 | vitis-13 | C2_At5g26880 | C2_At2g38730 | 19 668 160 | 23 137 507 | 3 469 347 | 4 | 4 |
| 10 | B | 205 | 223 | 17 | C2_At1g48050 | C2_At2g45620 | vitis-15 | C2_At1g48050 | C2_At2g46370 | 15 066 302 | 18 287 887 | 3 221 585 | 9 | 5 |
| 11 | C | 17 | 22 | 5 | C2_At4g15520 | C2_At1g05385 | vitis-5 | C2_At4g15520 | C2_At1g04190 | 4 006 420 | 5 592 417 | 1 585 997 | 4 | 3 |
| 12 | C | 38 | 54 | 16 | C2_At3g23490 | C2_At1g04690 | vitis-5 | C2_At3g23400 | C2_At1g04690 | 8 615 903 | 11 503 736 | 2 887 833 | 6 | 4 |
| 13 | D | 18 | 25 | 7 | C2_At1g79975 | C2_At3g18430 | vitis-9 | C2_At1g79975 | C2_At3g18430 | 4 305 431 | 8 720 941 | 4 415 510 | 7 | 4 |
| 14 | D | 49 | 57 | 7 | C2_At5g41040 | C2_At3g48610 | vitis-17 | C2_At5g41040 | C2_At5g63380 | 697 328 | 4 731 810 | 4 034 482 | 6 | 5 |
| 15 | E | 32 | 78 | 46 | C2_At2g32970 | C2_At2g38020 | vitis-12 | C2_At3g58790 | C2_At5g48300 | 263 945 | 10 197 511 | 9 933 566 | 18 | 14 |
| 16 | E | 81 | 100 | 19 | C2_At1g78600 | C2_At1g55870 | vitis-19 | C2_At1g78600 | C2_At1g55670 | 368 650 | 10 465 992 | 10 097 342 | 15 | 10 |
| 17 | F | 0 | 34 | 34 | C2_At3g08710 | C2_At5g32450 | vitis-8 | C2_At5g32450 | C2_At2g36930 | 3 358 003 | 18 994 732 | 15 636 729 | 15 | 9 |
| 18 | F | 36 | 59 | 23 | C2_At5g20350 | C2_At3g26060 | vitis-11 | C2_At3g26060 | C2_At5g20350 | 554 403 | 6 226 389 | 5 671 986 | 17 | 13 |
| 19 | G | 14 | 24 | 10 | C2_At5g40950 | C2_At5g39410 | vitis-14 | C2_At5g40950 | C2_At5g15410 | 25 610 981 | 29 993 772 | 4 382 791 | 11 | 7 |
| 20 | G | 48 | 56 | 7 | C2_At3g51010 | C2_At1g77370 | vitis-4 | C2_At5g66530 | C2_At4g35560 | 18 532 611 | 20 811 228 | 2 278 617 | 12 | 7 |
| 21 | H | 106 | 115 | 9 | C2_At1g64150 | C2_At2g45530 | vitis-2 | C2_At1g63970 | C2_At4g11570 | 479 584 | 3 020 649 | 2 541 065 | 5 | 5 |
| 22 | I | 6 | 8 | 2 | C2_At3g02870 | C2_At4g21770 | vitis-10 | C2_At4g21770 | C2_At3g02870 | 358 692 | 1 241 259 | 882 567 | 3 | 3 |
| 23 | I | 58 | 65 | 7 | C2_At4g21120 | C2_At1g29520 | vitis-10 | C2_At1g29520 | C2_At4g21120 | 4 614 182 | 7 994 682 | 3 380 500 | 4 | 3 |
| 24 | I | 74 | 83 | 9 | C2_At2g16920 | C2_At4g35250 | vitis-3 | C2_At2g16920 | C2_At4g35250 | 1 788 077 | 2 599 414 | 811 337 | 4 | 3 |
| 25 | J | 5 | 24 | 19 | C2_At1g30755 | C2_At3g03990 | vitis-18 | C2_At3g03990 | C2_At1g30755 | 7 602 554 | 12 401 321 | 4 798 767 | 16 | 12 |
| 26 | J | 34 | 58 | 24 | C2_At1g35720 | C2_At4g08230 | vitis-18 | C2_At1g35720 | C2_At4g08230 | 3 327 832 | 6 317 697 | 2 989 865 | 9 | 5 |
| 27 | J | 67 | 74 | 7 | C2_At1g21780 | C2_At1g77250 | vitis-18 | C2_At1g77250 | C2_At1g44575 | 2 150 284 | 2 907 044 | 756 760 | 4 | 4 |
| 28 | K | 39 | 60 | 21 | C2_At2g01110 | C2_At1g26520 | vitis-1 | C2_At1g26520 | C2_At2g01110 | 689 178 | 4 015 770 | 3 326 592 | 9 | 6 |
| 29 | K | 63 | 76 | 14 | C2_At1g69420 | C2_At1g67730 | vitis-1 | C2_At1g67730 | C2_At1g69420 | 6 671 460 | 13 673 702 | 7 002 242 | 10 | 6 |

Table S3. Syntenic blocks between coffee tree Linkage Groups and the pseudo-chromosomes of the grapevine genome.
