# Supplementary material for: Ancestral synteny shared between distantly-related plant species from the asterid (Coffea canephora and Solanum Sp.) and rosid (Vitis vinifera) clades
Source: BMC Genomics. 2012 Mar 20;13:103. doi: 10.1186/1471-2164-13-103 (PMC3372433; doi:10.1186/1471-2164-13-103)
Supplement: Additional file 5 — Table S4 Syntenic Blocks between Tomato Chromosomes and the Pseudo-Chromosomes of the Grapevine Genome. [file 1471-2164-13-103-S5.DOC]

**Supporting Information** Guyot *et al*., “Ancestral Synteny Shared between Distantly-Related Plant Species from the Asterid (Coffea canephora and Solanum sp.) and Rosid (Vitis vinifera) Clades”

| **CSS #** | **Tomato LG** | **Tomato CSS position (cM)** | **Tomato CSS**  **position**  **(cM)** | **Tomato CSS size (cM)** | **COS** | **COS** | **Vitis pseudochr.** | **COS** | **COS** | **Vitis CSS position(bp)** | **Vitis CSS position (bp)** | **Vitis CSS size (bp)** | **Coffee COSII per block** | **Coffee COSII synthenic per CSS** |
| --- | --- | --- | --- | --- | --- | --- | --- | --- | --- | --- | --- | --- | --- | --- |
| 1 | 1 | 30 | 47 | 17 | C2_At3g06580 | C2_At4g15520 | vitis-5 | C2_At3g06580 | C2_At3g06050 | 490 792 | 4 545 644 | 4 054 852 | 21 | 6 |
| 2 | 1 | 44 | 48 | 4 | C2_At3g63490 | C2_At3g52120 | vitis-13 | C2_At3g63490 | C2_At3g52120 | 21 630 441 | 23 150 478 | 1 520 037 | 14 | 5 |
| 3 | 1 | 50 | 88 | 38 | C2_At1g02090 | C2_At5g41760 | vitis-15 | C2_At3g60300 | C2_At4g00560 | 11 900 471 | 20 207 051 | 8 306 580 | 16 | 12 |
| 4 | 1 | 99 | 124 | 25 | C2_At3g04780 | C2_At5g26680 | vitis-14 | C2_At5g26680 | C2_At3g04780 | 177 393 | 6 803 145 | 6 625 752 | 20 | 9 |
| 5 | 1 | 127 | 136 | 8 | C2_At5g49880 | C2_At1g25260 | vitis-12 | C2_At1g25260 | C2_At5g49880 | 16 842 992 | 21 979 272 | 5 136 280 | 6 | 5 |
| 6 | 1 | 146 | 165 | 19 | C2_At3g19630 | C2_At1g16210 | vitis-3 | C2_At4g34700 | C2_At3g19630 | 615 832 | 7 298 343 | 6 682 511 | 14 | 11 |
| 7 | 2 | 37 | 49 | 12 | C2_At4g20410 | C2_At5g45760 | vitis-10 | C2_At5g45760 | C2_At5g44560 | 2 952 277 | 11 913 259 | 8 960 982 | 10 | 5 |
| 8 | 2 | 83 | 89 | 6 | C2_At2g18030 | C2_At4g36530 | vitis-4 | C2_At5g66530 | C2_At4g38630 | 18 532 027 | 20 149 973 | 1 617 946 | 9 | 4 |
| 9 | 2 | 91 | 102 | 11 | C2_At3g01480 | C2_At3g01180 | vitis-14 | C2_At3g55250 | C2_At3g01180 | 27 790 687 | 29 588 449 | 1 797 762 | 10 | 5 |
| 10 | 3 | 45 | 55 | 10 | C2_At5g24120 | C2_At5g23940 | vitis-16 | C2_At5g23880 | C2_At5g24120 | 17 553 039 | 19 490 964 | 1 937 925 | 7 | 4 |
| 11 | 3 | 72 | 77 | 5 | C2_At1g64770 | C2_At3g10220 | vitis-8 | C2_At3g10220 | C2_At3g57680 | 19 373 846 | 21 400 292 | 2 026 446 | 15 | 3 |
| 12 | 3 | 73 | 73 | 0 | C2_At5g17560 | C2_At5g17170 | vitis-16 | C2_At5g17560 | C2_At5g17170 | 265 237 | 2 777 366 | 2 512 129 | 4 | 3 |
| 13 | 3 | 101 | 122 | 21 | C2_At5g13050 | C2_At1g74470 | vitis-17 | C2_At5g23060 | C2_At3g47990 | 4 731 998 | 9 278 190 | 4 546 192 | 9 | 6 |
| 14 | 3 | 123 | 130 | 7 | C2_At1g80170 | C2_At1g80460 | vitis-9 | C2_At1g80170 | C2_At1g80460 | 6 292 330 | 8 308 527 | 2 016 197 | 5 | 3 |
| 15 | 3 | 141 | 145 | 4 | C2_At1g18270 | C2_At3g18520 | vitis-17 | C2_At1g18270 | C2_At3g18520 | 1 768 539 | 4 213 760 | 2 445 221 | 4 | 4 |
| 16 | 3 | 149 | 171 | 23 | C2_At1g79910 | C2_At3g14075 | vitis-9 | C2_At3g14075 | C2_At1g79910 | 7 308 | 4 147 946 | 4 140 638 | 10 | 5 |
| 17 | 4 | 55 | 57 | 1 | C2_At3g54770 | C2_At3g07950 | vitis-13 | C2_At3g54770 | C2_At2g28880 | 4 762 759 | 5 499 513 | 736 754 | 5 | 3 |
| 18 | 4 | 66 | 136 | 70 | C2_At1g77250 | C2_At1g42990 | vitis-18 | C2_At1g42990 | C2_At2g45730 | 423 103 | 12 171 427 | 11 748 324 | 25 | 17 |
| 19 | 5 | 7 | 19 | 12 | C2_At1g60200 | C2_At1g14790 | vitis-1 | C2_At1g14790 | C2_At1g07040 | 5 631 115 | 10 786 076 | 5 154 961 | 9 | 4 |
| 20 | 5 | 37 | 56 | 19 | C2_At2g01110 | C2_At1g26520 | vitis-1 | C2_At2g01110 | C2_At1g26520 | 689 732 | 4 015 770 | 3 326 038 | 10 | 7 |
| 21 | 5 | 64 | 69 | 5 | C2_At3g26085 | C2_At1g27980 | vitis-1 | C2_At3g26085 | C2_At1g27980 | 19 157 723 | 21 481 422 | 2 323 699 | 4 | 3 |
| 22 | 6 | 19 | 24 | 5 | C2_At3g56040 | C2_At3g11210 | vitis-13 | C2_At5g05690 | C2_At3g56040 | 361 153 | 826 205 | 465 052 | 3 | 3 |
| 23 | 6 | 37 | 40 | 3 | C2_At1g21640 | C2_At1g44760 | vitis-18 | C2_At1g21640 | C2_At1g44835 | 2 342 296 | 3 223 227 | 880 931 | 5 | 5 |
| 24 | 6 | 48 | 56 | 8 | C2_At1g73885 | C2_At5g62530 | vitis-17 | C2_At1g73885 | C2_At5g07960 | 3 759 857 | 7 900 118 | 4 140 261 | 4 | 3 |
| 25 | 7 | 0 | 24 | 24 | C2_At5g20350 | C2_At1g19140 | vitis-11 | C2_At1g19140 | C2_At4g30580 | 711 033 | 8 001 580 | 7 290 547 | 14 | 6 |
| 26 | 7 | 15 | 29 | 14 | C2_At2g26590 | C2_At4g31040 | vitis-11 | C2_At4g31040 | C2_At2g06925 | 14 271 362 | 15 801 121 | 1 529 759 | 9 | 3 |
| 27 | 7 | 38 | 49 | 11 | C2_At4g26680 | C2_At2g38020 | vitis-12 | C2_At3g58790 | C2_At4g26680 | 263 963 | 9 350 395 | 9 086 432 | 15 | 10 |
| 28 | 7 | 54 | 68 | 14 | C2_At1g53670 | C2_At3g15430 | vitis-19 | C2_At1g53670 | C2_At3g15430 | 153 854 | 3 261 235 | 3 107 381 | 6 | 5 |
| 29 | 7 | 80 | 110 | 30 | C2_At4g26750 | C2_At1g55670 | vitis-19 | C2_At4g26750 | C2_At1g55670 | 7 305 384 | 10 466 017 | 3 160 633 | 10 | 6 |
| 30 | 8 | 20 | 41 | 21 | C2_At4g33090 | C2_At5g25630 | vitis-4 | C2_At4g33090 | C2_At5g25630 | 533 270 | 7 024 484 | 6 491 214 | 22 | 12 |
| 31 | 8 | 50 | 87 | 37 | C2_At1g32220 | C2_At4g11570 | vitis-2 | C2_At1g63980 | C2_At4g19003 | 503 854 | 9 983 433 | 9 479 579 | 19 | 8 |
| 32 | 9 | 0 | 17 | 17 | C2_At5g01510 | C2_At2g32600 | vitis-8 | C2_At2g37025 | C2_At2g41680 | 12 778 934 | 22 143 625 | 9 364 691 | 8 | 8 |
| 33 | 9 | 35 | 43 | 8 | C2_At3g52610 | C2_At5g02230 | vitis-8 | C2_At2g37500 | C2_At3g52610 | 14 291 290 | 17 140 102 | 2 848 812 | 4 | 3 |
| 34 | 9 | 51 | 61 | 10 | C2_At2g48120 | C2_At2g47590 | vitis-7 | C2_At3g63190 | C2_At2g47590 | 1 301 588 | 3 261 764 | 1 960 176 | 10 | 6 |
| 35 | 9 | 85 | 99 | 15 | C2_At3g23400 | C2_At3g24010 | vitis-5 | C2_At3g23400 | C2_At3g24010 | 8 615 386 | 10 908 495 | 2 293 109 | 6 | 5 |
| 36 | 10 | 0 | 0 | 0 | C2_At3g20390 | C2_At3g13235 | vitis-19 | C2_At3g20390 | C2_At3g13235 | 6 548 160 | 9 732 809 | 3 184 649 | 3 | 3 |
| 37 | 10 | 37 | 39 | 3 | C2_At5g01010 | C2_At1g32070 | vitis-8 | C2_At5g01990 | C2_At1g32070 | 248 855 | 879 154 | 630 299 | 4 | 3 |
| 38 | 11 | 10 | 21 | 11 | C2_At3g52220 | C2_At3g54840 | vitis-13 | C2_At3g54840 | C2_At3g52220 | 6 234 279 | 11 622 968 | 5 388 689 | 5 | 4 |
| 39 | 11 | 40 | 41 | 1 | C2_At1g44446 | C2_At4g08230 | vitis-18 | C2_At1g44446 | C2_At4g08230 | 2 882 472 | 3 327 832 | 445 360 | 4 | 4 |
| 40 | 11 | 47 | 58 | 11 | C2_At3g20870 | C2_At2g28800 | vitis-6 | C2_At3g20870 | C2_At2g28800 | 2 291 208 | 5 409 680 | 3 118 472 | 13 | 3 |
| 41 | 11 | 61 | 73 | 12 | C2_At3g44880 | C2_At2g27450 | vitis-6 | C2_At5g22940 | C2_At2g27450 | 18 035 528 | 20 567 562 | 2 532 034 | 9 | 6 |
| 42 | 11 | 76 | 82 | 6 | C2_At1g56450 | C2_At2g28250 | vitis-6 | C2_At2g28250 | C2_At1g56450 | 3 272 346 | 3 973 068 | 3 272 346 | 5 | 4 |
| 43 | 11 | 97 | 103 | 6 | C2_At5g58490 | C2_At5g59960 | vitis-6 | C2_At2g28490 | C2_At5g58490 | 4 035 705 | 7 900 773 | 4 035 705 | 3 | 3 |
| 44 | 12 | 14 | 34 | 20 | C2_At4g28830 | C2_At2g06005 | vitis-11 | C2_At2g06005 | C2_At3g25910 | 894 038 | 4 186 306 | 3 292 268 | 9 | 6 |
| 45 | 12 | 105 | 115 | 10 | C2_At1g48310 | C2_At3g17000 | vitis-5 | C2_At3g06610 | C2_At1g48310 | 690 293 | 2 759 967 | 2 069 674 | 3 | 3 |

**Table S4. Syntenic blocks between tomato chromosomes and the pseudo-chromosomes of the grapevine genome**
