# Supplementary material for: Ancestral synteny shared between distantly-related plant species from the asterid (Coffea canephora and Solanum Sp.) and rosid (Vitis vinifera) clades
Source: BMC Genomics. 2012 Mar 20;13:103. doi: 10.1186/1471-2164-13-103 (PMC3372433; doi:10.1186/1471-2164-13-103)
Supplement: Additional file 6 — Figure S2 Detailed Examples of Macrosynteny between the Coffee Tree, the Tomato and the Grapevine. [file 1471-2164-13-103-S6.DOC]

**Supporting Information** Guyot *et al*., “Ancestral Synteny Shared between Distantly-Related Plant Species from the Asterid (*Coffea canephora* and *Solanum* sp.) and Rosid (*Vitis vinifera*) Clades”

**
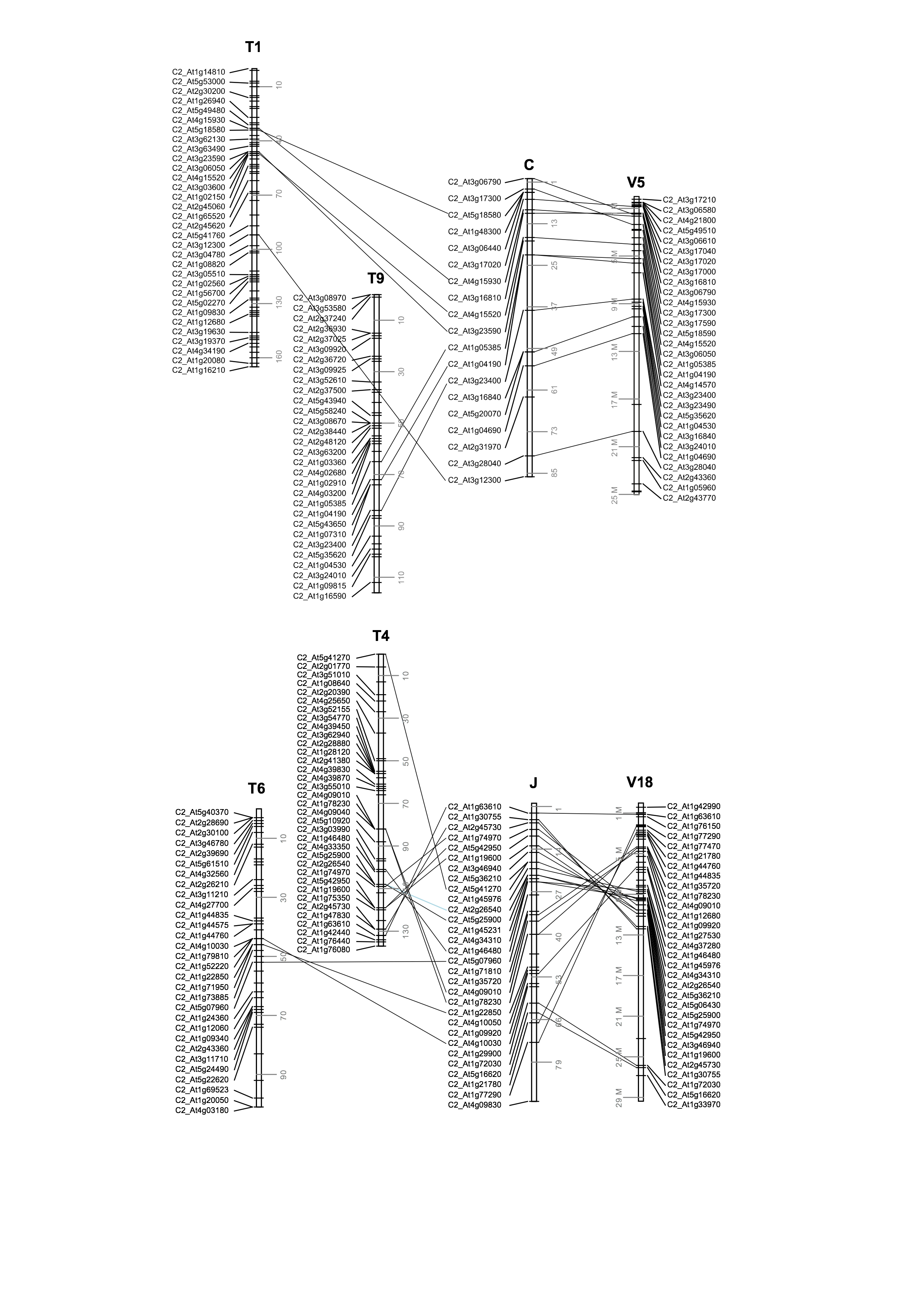
**

**
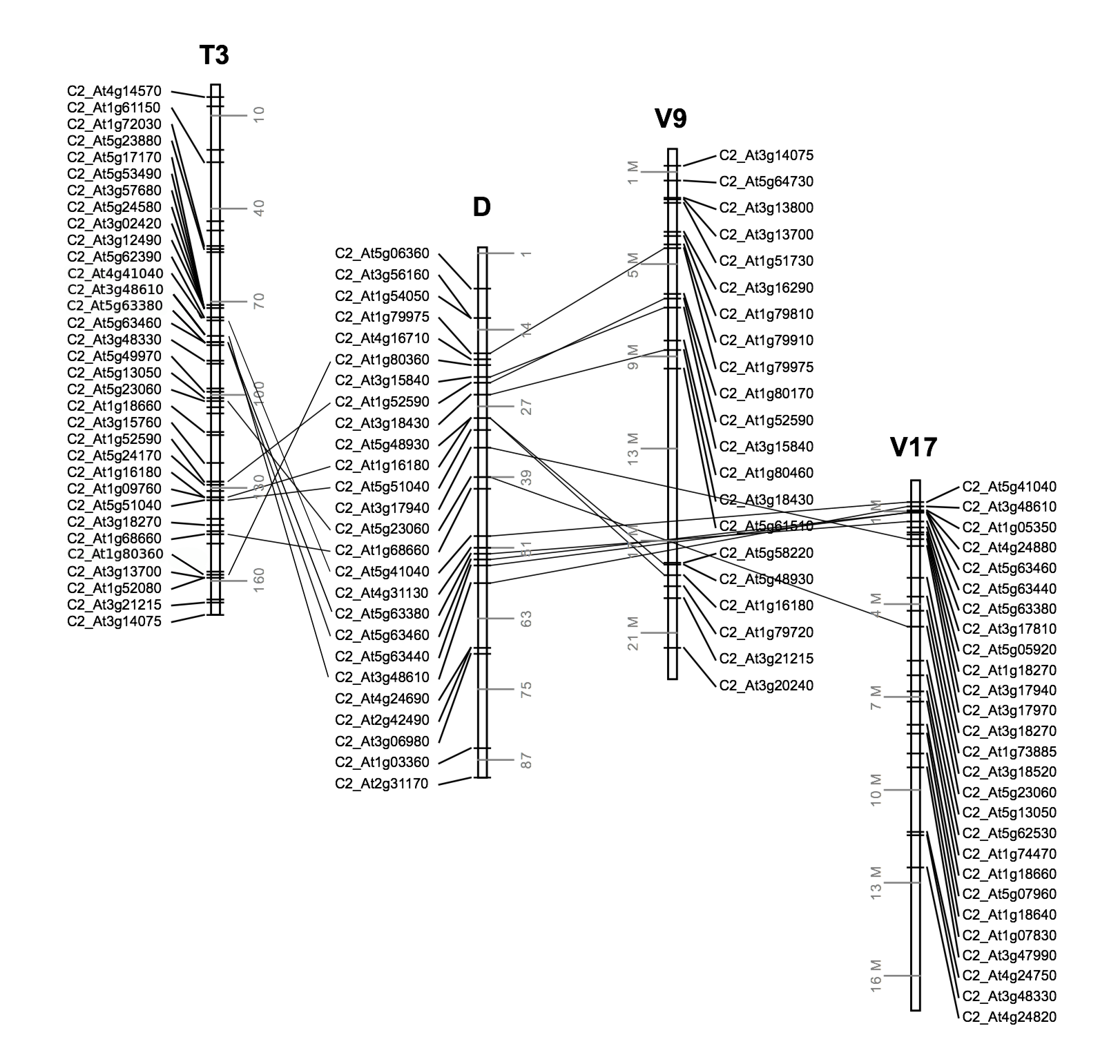
**


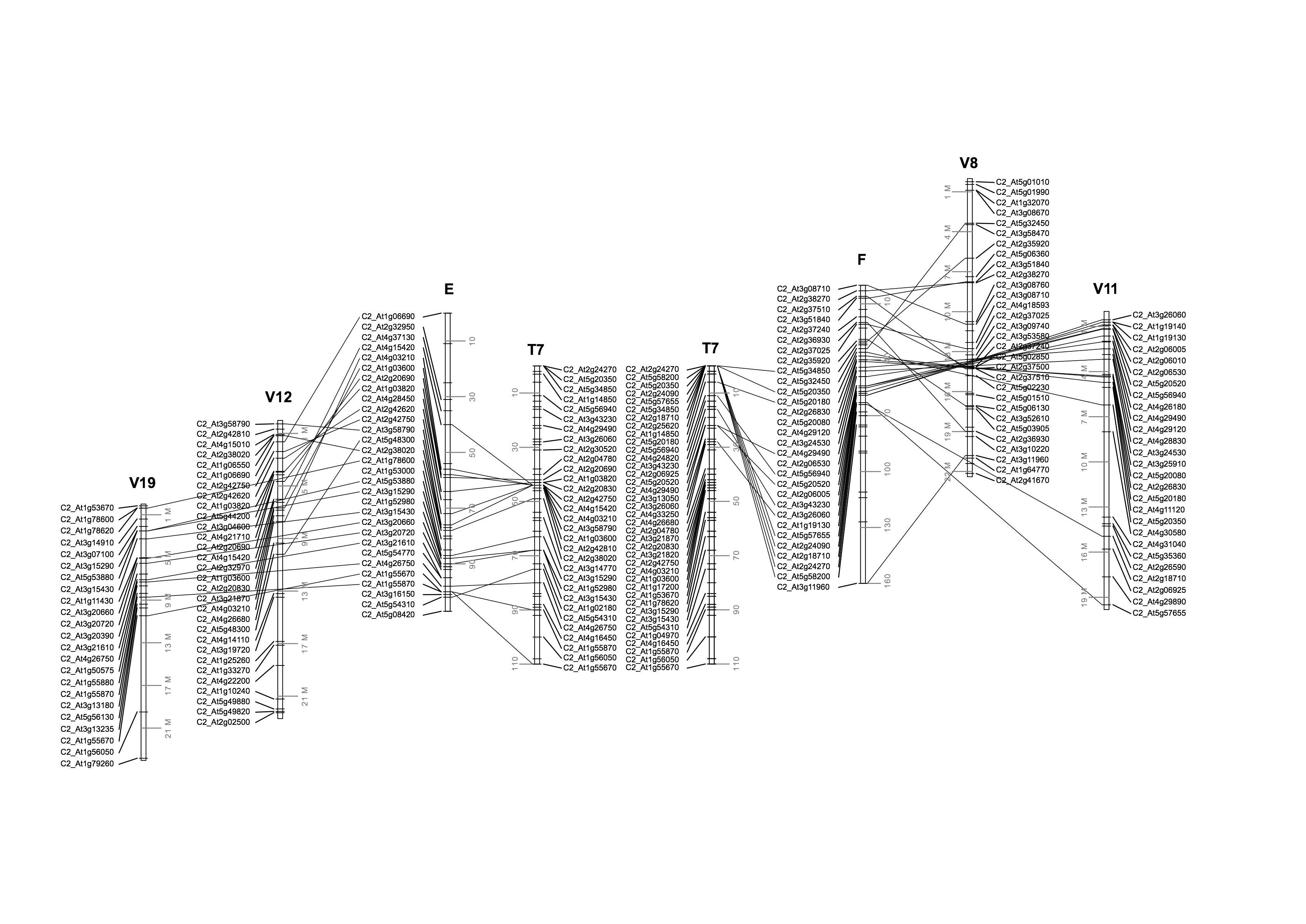


Fig. S2. Detailed examples of macrosynteny between the coffee tree, the tomato and the grapevine.

Each vertical bar represents linkage groups of coffee (letters) and tomato (T), and grapevine (V) chromosomes. COSII names are shown on one side. Centimorgan values are indicated for LGs and Megabase positions (Mb) are given for chromosomes. Lines connecting COSII markers between different LG or chromosomes represent conserved markers. All comparative maps are available via our website (http://moccadb-test.mpl.ird.fr/).
