# Supplementary material for: Ancestral synteny shared between distantly-related plant species from the asterid (Coffea canephora and Solanum Sp.) and rosid (Vitis vinifera) clades
Source: BMC Genomics. 2012 Mar 20;13:103. doi: 10.1186/1471-2164-13-103 (PMC3372433; doi:10.1186/1471-2164-13-103)
Supplement: Additional file 7 — Table S5 List of Identified Genes in the Coffee Tree (C. canephora) BAC clone 111O18. [file 1471-2164-13-103-S7.DOC]

**Supporting Information** Guyot *et al*., “Ancestral Synteny Shared between Distantly-Related Plant Species from the Asterid (*Coffea canephora* and *Solanum* sp.) and Rosid (*Vitis vinifera*) Clades”


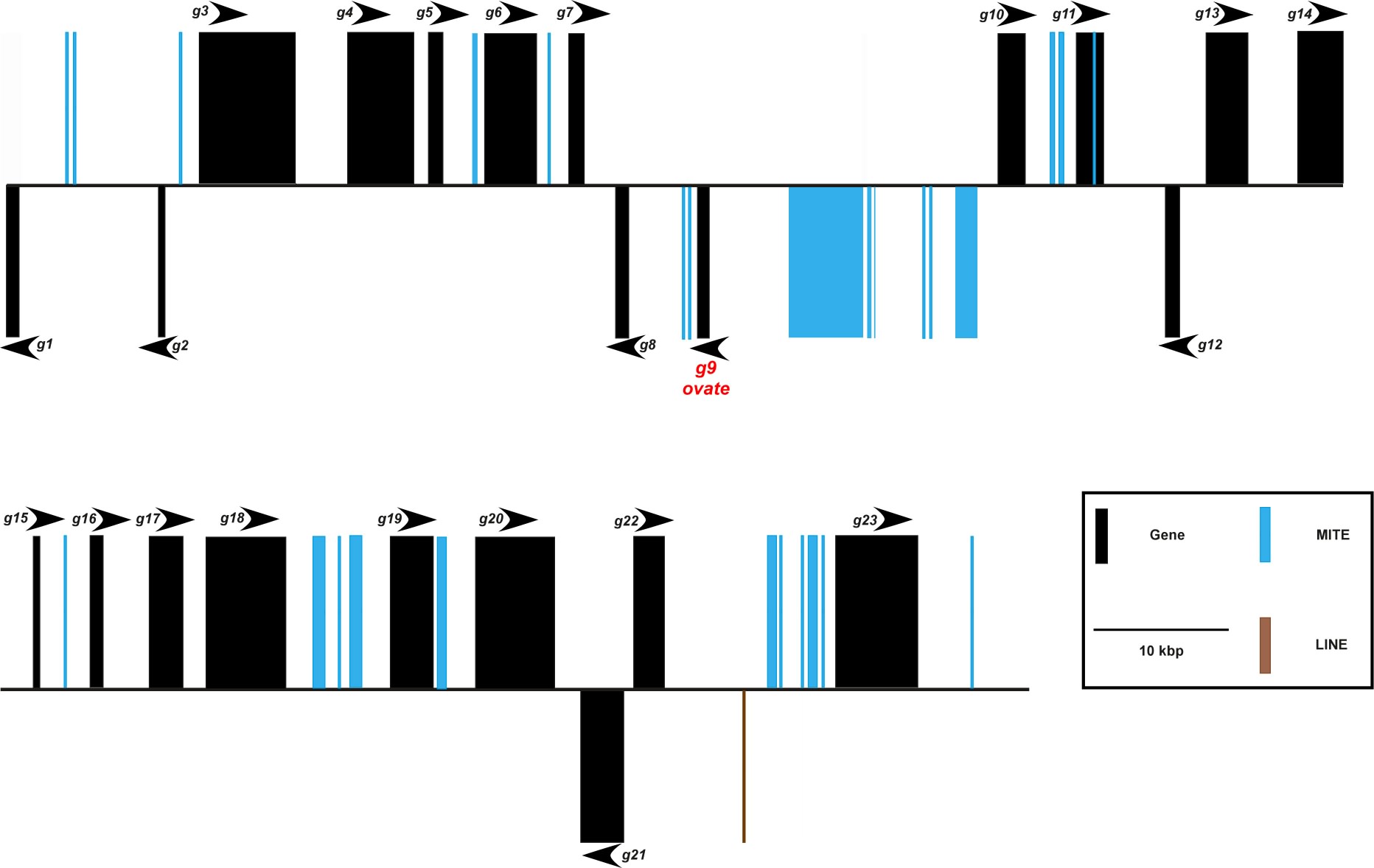


**Fig. S3. Physical map and annotation of the 174,135 bp of the *C. canephora* BAC clone 111O18.**

Black, brown and blue boxes represent genes, retrotransposons (LINE) and transposons, respectively. Genes are labeled as listed in Table S6. A black arrow indicates orientation of genes.
