# Supplementary material for: Ancestral synteny shared between distantly-related plant species from the asterid (Coffea canephora and Solanum Sp.) and rosid (Vitis vinifera) clades
Source: BMC Genomics. 2012 Mar 20;13:103. doi: 10.1186/1471-2164-13-103 (PMC3372433; doi:10.1186/1471-2164-13-103)
Supplement: Additional file 9 — Table S6 List of orthologous predicted genes in the ovate regions of C. canephora, Solanaceae and the grapevine genome. [file 1471-2164-13-103-S9.DOC]

**Supporting Information** Guyot *et al*., “Ancestral Synteny Shared between Distantly-Related Plant Species from the Asterid (*Coffea canephora* and *Solanum* sp.) and Rosid (*Vitis vinifera*) Clades”


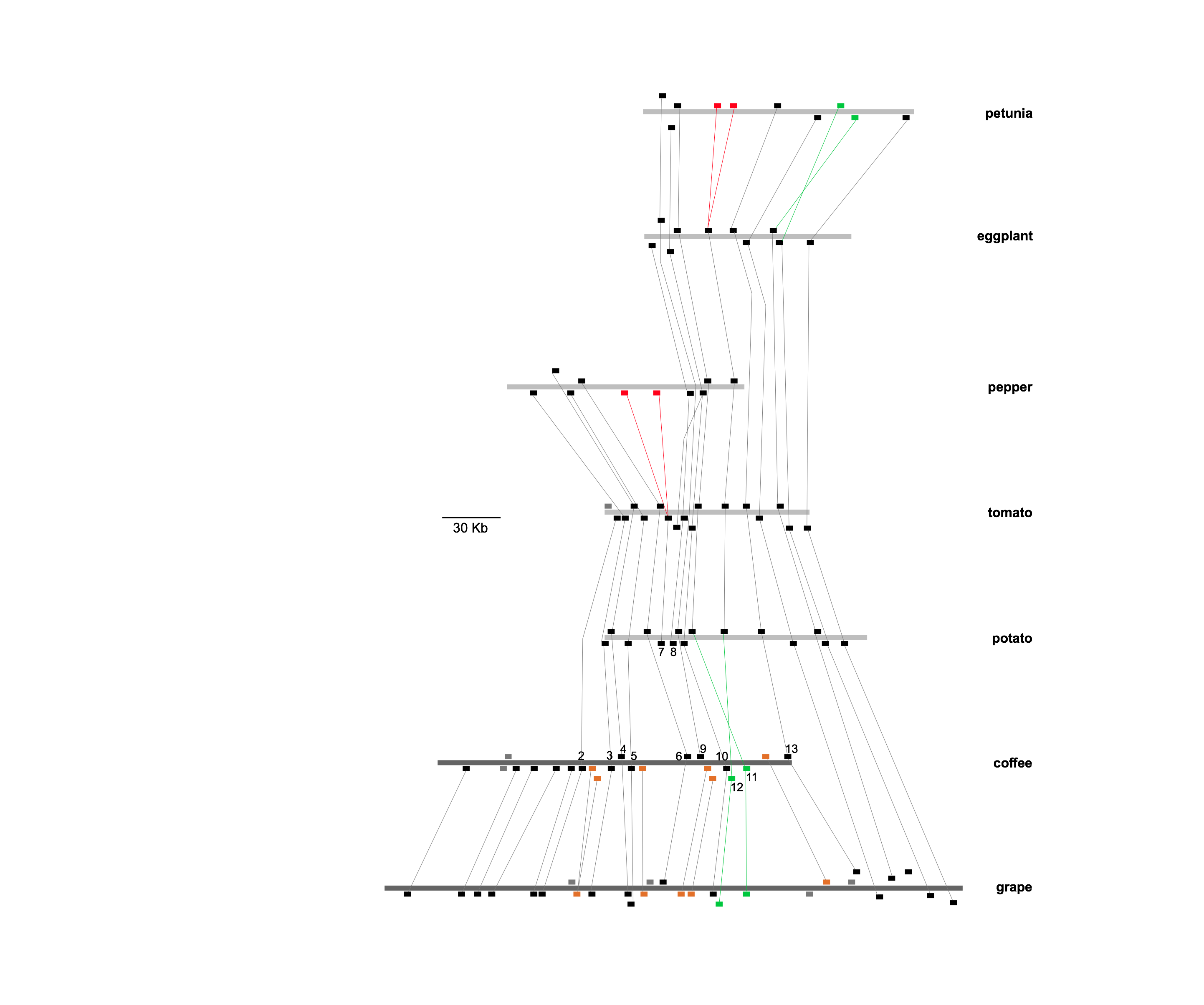


Fig. S4.  Scale map of the microsynteny between five species in Solanaceae*,* *Coffea canephora* (Rubiaceae) and *Vitis vinifera* (Vitaceae).

The sequenced segments of the potato (*Solanum bulbocastanum, 135 kb*), tomato (*Solanum lycopersicum, 105 kb*), pepper (*Capsicum annuum, 122 kb*), eggplant (*Solanum melongena, 106 kb*) and petunia (*Petunia inflata, 139 kb*) (28) were compared to orthologous segments in coffee (*Coffea canephora*, BAC Clone 111O18, 174 kb) and grapevine (*Vitis* genome, Chromosome 4, Positions 17,954-18,168 Mb; 234kb). Arrows symbolize orientation of predicted coding regions. Letters and numbers linked to arrows indicate orthologous gene families as defined in **Table S6**. Lines link coding regions between conserved syntenic segments. Duplicated genes are represented in red, while inverted genes are represented in green. Genes conserved between coffee and grape but absent in orthologous locations in the five *Solanaceae* species were indicated in orange. Genes present in only one species are represented in grey. The different segments represented here are to scale.
